# A Tutorial Review of Labeling Methods in Mass Spectrometry-Based Quantitative Proteomics

**DOI:** 10.1021/acsmeasuresciau.4c00007

**Published:** 2024-04-15

**Authors:** Zicong Wang, Peng-Kai Liu, Lingjun Li

**Affiliations:** † School of Pharmacy, University of Wisconsin—Madison, Madison, Wisconsin 53705, United States; ‡ Biophysics Graduate program, University of Wisconsin—Madison, Madison, Wisconsin 53705, United States; § Department of Chemistry, University of Wisconsin—Madison, Madison, Wisconsin 53706, United States; ∥ Lachman Institute for Pharmaceutical Development, School of Pharmacy, University of Wisconsin—Madison, Madison, Wisconsin 53705, United States; ⊥ Wisconsin Center for NanoBioSystems, School of Pharmacy, University of Wisconsin—Madison, Madison, Wisconsin 53705, United States

**Keywords:** Stable isotope labeling, Mass spectrometry, Quantitative proteomics, Peptides and proteins, Reporter ion-based quantitation, Isotopic and isobaric tagging, Protein quantitation, Systems biology, Posttranslational modifications, Tutorial review

## Abstract

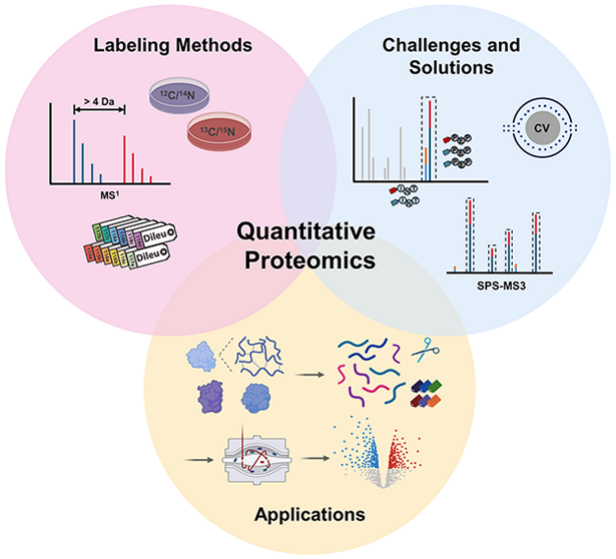

Recent advancements in mass spectrometry (MS) have revolutionized quantitative proteomics, with multiplex isotope labeling emerging as a key strategy for enhancing accuracy, precision, and throughput. This tutorial review offers a comprehensive overview of multiplex isotope labeling techniques, including precursor-based, mass defect-based, reporter ion-based, and hybrid labeling methods. It details their fundamental principles, advantages, and inherent limitations along with strategies to mitigate the limitation of ratio-distortion. This review will also cover the applications and latest progress in these labeling techniques across various domains, including cancer biomarker discovery, neuroproteomics, post-translational modification analysis, cross-linking MS, and single-cell proteomics. This Review aims to provide guidance for researchers on selecting appropriate methods for their specific goals while also highlighting the potential future directions in this rapidly evolving field.

## 1 Introduction

Mass spectrometry (MS) has been a cornerstone in the field of proteomics, offering unparalleled insights into complex protein information within biological systems. Over the past decades, MS-based proteomics has facilitated accurate genome-wide quantification of protein changes across multiple biological states in a single experiment.^1,2^ This evolution is largely attributed to the advancements in labeling techniques, which have markedly enhanced the precision, accuracy, and throughput of quantitative proteomic studies.^3,4^ Consequently, these labeling techniques have broadened the scope and capabilities of proteomic research, catalyzing significant progress in key applications such as protein structural elucidation,^5−7^ biomarker discovery,^8−12^ and drug development.^13−16^

**Figure 1:**
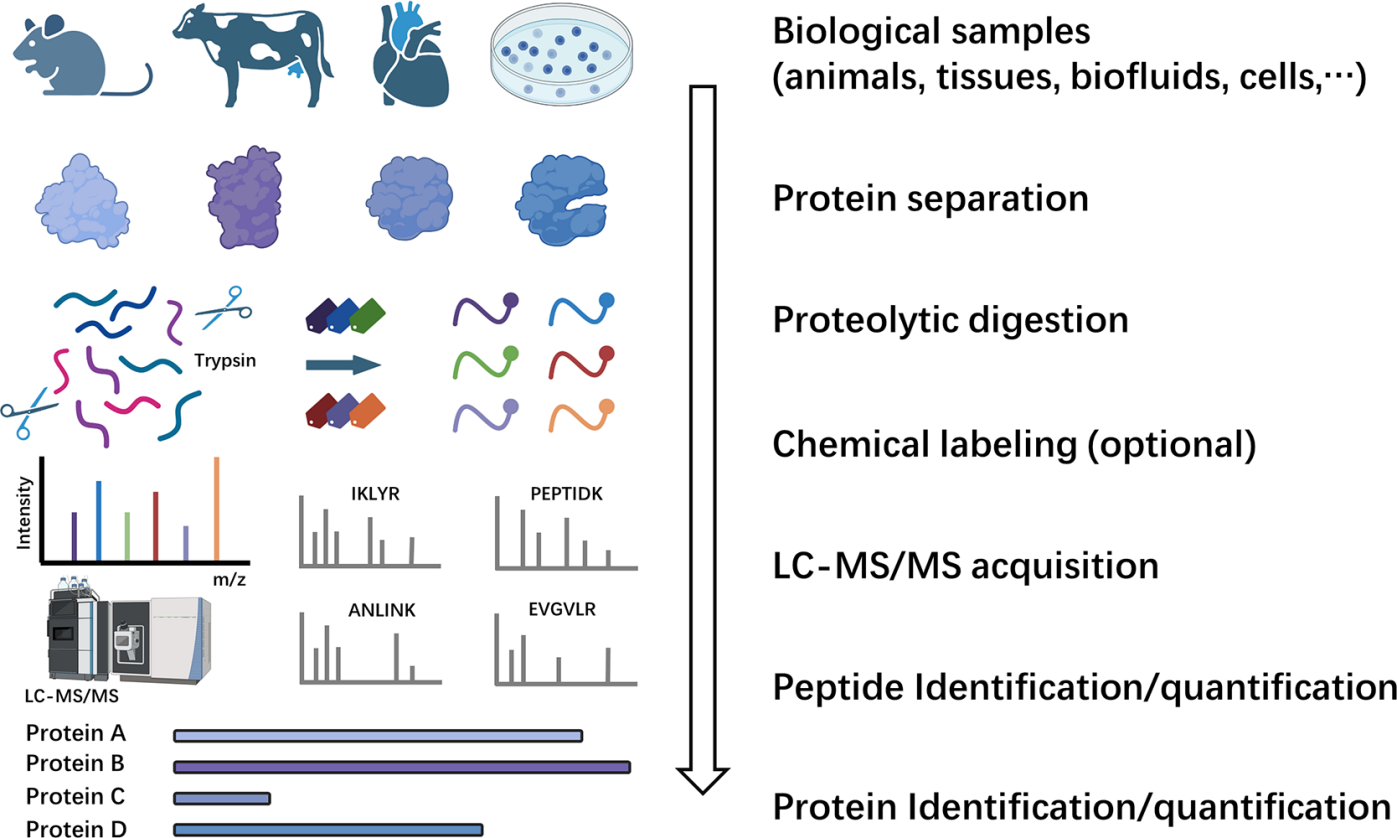
Conceptual workflow of bottom-up proteomics. Initially, proteins are extracted from biological samples and subsequently digested into peptides. Through chemical labeling strategies, stable isotopes are incorporated into peptides. The peptides are then analyzed using an LC-MS/MS system. The MS/MS mass spectra are analyzed via database search algorithms to identify peptides matching. Subsequently, the identified peptides in the sample are mapped to their source proteins, facilitating the accurate identification and quantification of proteins from complex mixtures. Figure created with BioRender.com.^234^

In the MS-based proteomics field, the predominant methodology employed is the bottom-up strategy.^2,3,17^ This involves initially breaking down proteins into peptides through proteolytic digestion (Figure 1). This workflow begins with protein extraction, denaturation, reduction, and alkylation from biological samples to prepare them for enzymatic digestion. Enzymatic digestion is typically performed with trypsin, which cleaves proteins at the C-terminus of lysine and arginine residues with high specificity.^18^ Following digestion, peptides undergo rigorous cleanup processes, such as solid-phase extraction, to remove contaminants and, optionally, enrichment for targeted modification studies. The refined peptide mixture is separated via high-performance liquid chromatography (HPLC), primarily reversed-phase HPLC (RPLC) based on hydrophobicity, and then introduced into the mass spectrometer using an electrospray ionization (ESI) source.^17^ In MS, the mass-to-charge ratios and signal intensities of ionized peptides and their corresponding fragmentation products are measured. Bioinformatics tools process these fragmentation spectrum data against theoretical spectra generated from *in silico* digestion of protein databases, with strict statistical analysis to control the false discovery rate (FDR) and ensure result reliability.^19,20^ Subsequently, the identified peptides in the sample are mapped to their source proteins, facilitating the identification of these related proteins.

Mass spectrometry is not intrinsically quantitative.^2^ The peak height or area in a mass spectrum does not accurately reflect a peptide’s abundance in the sample due to variations in peptide ionization efficiency and detectability.^21^ Obtaining quantitative information necessitates significant additional effort. Initially, label-free quantitation methods in MS-based proteomics were developed. It relied on matching peaks across different LC-MS runs and comparing the intensities of the peptide ions or spectrum counts.^22−25^ A significant challenge with this approach is run-to-run variability, which stems from differences in chromatographic separation, ionization efficiency, and instrument performance, all contributing to inaccurate quantification. Label-free methods also face the issue of missing values, with a substantial portion of peptides undetected across replicates, complicating result comparisons across experimental runs.^26^

To address these limitations, stable isotope labeling techniques were introduced.^27^ These techniques involve the incorporation of stable isotopes into different samples, combining them for sample preparation and analysis, then distinguishing them in the MS based on their *m*/*z* difference. Isotopic labeling allows for the direct comparison of different samples within the same MS run, thereby reducing variability and improving quantitative accuracy.^25,28^

Labeling methods in quantitative proteomics can be categorized into various subcategories based on different criteria. These include quantitative ions (precursor ion-based quantification, reporter ion-based quantification, and hybrid methods), resolution requirements for distinguishing mass differences (high resolution required at precursor ion level, reporter ion level, or not required), and the methods of isotopic incorporation (chemical, enzymatic, or metabolic). This review aims to provide an overview of different labeling methods, focusing on their fundamental principles, advantages, and limitations. The review will also cover the applications and recent advancements of these labeling strategies, guiding researchers to choose suitable methods for their research goals and highlighting potential future directions in this rapidly evolving field.

## 2 Quantitative Labeling Methods

### 2.1 Precursor Ion-Based Quantification

**Figure 2:**
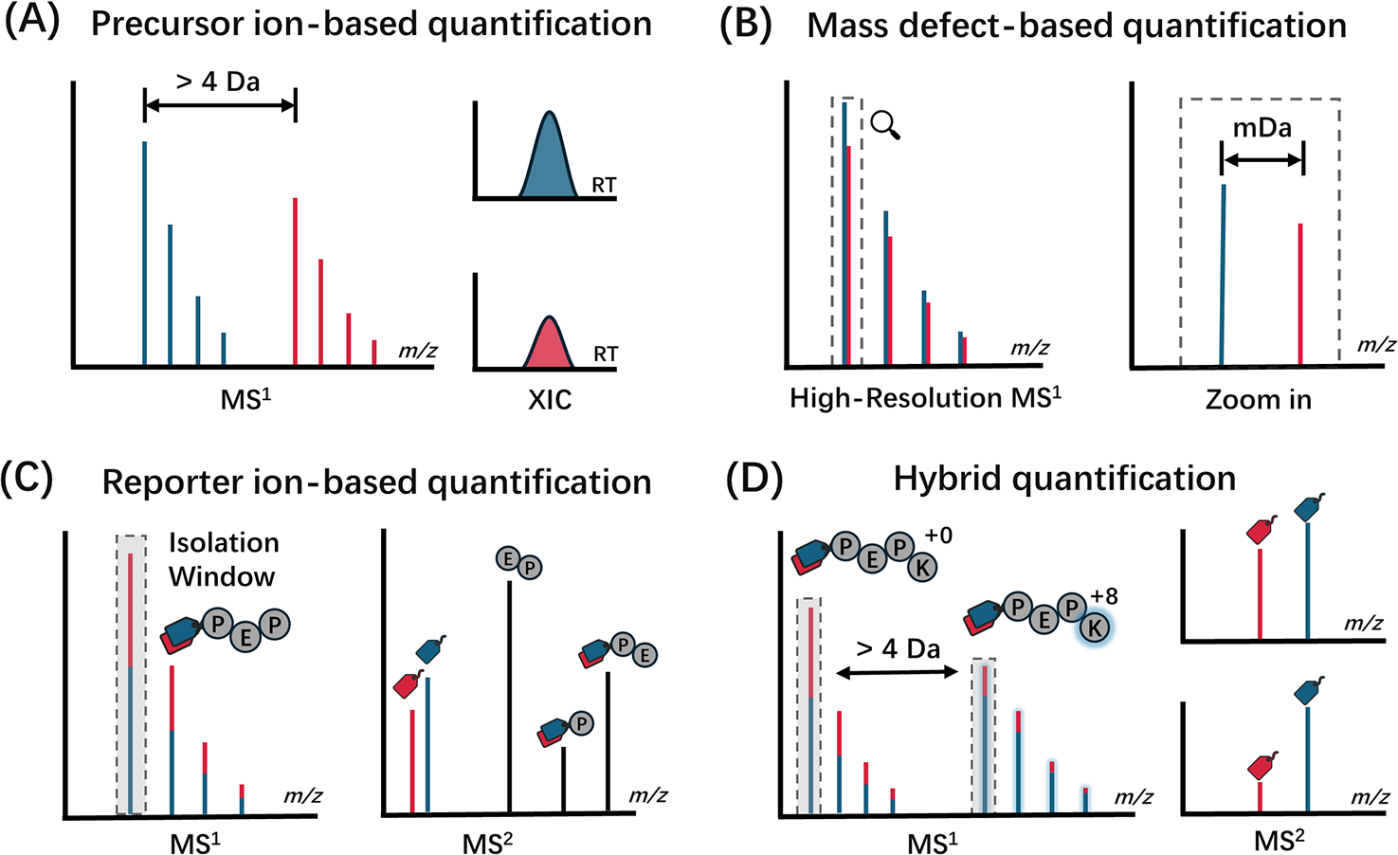
Graphical representation of quantitative labeling approaches in mass spectrometry. (A) Precursor ion-based quantification. Light and heavy labeled sample precursor ions are distinguished by a mass difference larger than 4 Da, Relative quantitation is achieved through the extracted ion chromatograms (XIC) for each labeled species. (B) Mass defect-based precursor ion quantification. This method exploits subtle mass difference introduced to each labeled species, reducing spectrum complexity, and enhancing multiplexing capability. (C) Reporter ion-based quantification. This method utilizes isobaric tags for labeling peptides from distinct samples then mixed them for MS/MS analysis. Labeled peptides will only generate a single peak in MS1 spectrum. Quantification is based on the intensity of their fragmentation-generated reporter ions. (D) Hybrid quantification. This method combines the principles of both light/heavy and isobaric tagging to enhance multiplexing capabilities.

Precursor ion-based quantitation stands as one of the earliest and most fundamental methods in proteomics.^29^ These methods differentiate between light and heavy labeled samples by a mass difference introduced by stable isotopes. This mass difference is normally greater than 4 Da to minimize isotopic envelope interference.^30^ Relative quantitation of these samples is achieved through comparing the extraction ion chromatograms from peptide precursor ions in MS1 spectra (Figure 2A).^25^ Depending on the method for introducing stable isotope labels, precursor ion quantitation techniques can be categorized as metabolic, enzymatic, and chemical labeling.

Metabolic labeling involves culturing cells or feeding animals with isotopically labeled amino acids, facilitating label incorporation at the cellular or protein level. This approach reduces variations introduced in sample preparation at an early stage, thereby enhancing the quantitative accuracy. Consequently, metabolic labeling was considered the gold standard in proteomics quantification.^31^

^15^N labeling is one of the earliest developed metabolic labeling methods.^32^ Its application in quantitation is limited due to the variable number of ^15^N labels in peptides, leading to a complex isotopic pattern in the resulting spectra. Amino acid stable isotope labeling in cell culture (SILAC) is a more widely used method.^33^ Stable isotopic labeled amino acids, typically lysine and arginine, are incorporated into cell culture media.^33−35^ After trypsin digestion, all peptides (except protein C-terminal ones) will have a predictable mass shift, simplifying data interpretation.^36^ SILAC is extensively used in studies of cell-derived biological processes,^37−41^ including single-cell analysis,^42^ and is adaptable to multicellular organisms by feeding labeled amino acids.^43−46^

Enzymatic labeling, with ^18^O labeling as the primary method, involves enzymatic digestion of proteins in ^18^O labeled water. This process will incorporate two ^18^O atoms into the C-terminal carboxyl groups of digested peptides.^47−49^ Although it has limited multiplexing capabilities of 2-plex, this method is suitable for a variety of proteomic samples and complements other labeling techniques.^29^

Chemical labeling introduces stable isotope labels by attaching various tags to the functional groups of peptides *in vitro*. The chemical labeling methods can be applied across a wide variety of samples including cells, tissues, and various bodily fluids.

The Isotope-Coded Affinity Tag (ICAT) was the first commercial reagent developed for proteomic quantification.^50,51^ It features an iodoacetyl group for reacting with cysteine’s thiol group. However, this strategy loses information for all peptides without cysteine. Subsequently, the dimethylation reaction of N-termini and lysine residues (α- and ε-amine groups) was introduced to quantitative proteomics.^52^ This reaction involves the formation of a Schiff base with formaldehyde and subsequent reduction to dimethylamine with cyanoborohydride. The dimethylation reaction has high efficiency in mild conditions and produces no significant byproducts.^53^ Furthermore, dimethylation improves the ionization efficiency of peptides since the formed tertiary amine exhibits enhanced ionization capabilities in electrospray.^54^ By utilizing different isotopic forms of reagents, triplex dimethylation can be achieved with 4 Da mass difference.^30^ Applying Lys-C enzyme for digestion, which results in each peptide having two reactive amine groups, allows for an increase in throughput to 5-plex labeling.^55^

There are also other amine-reactive chemical tags like mTMT,^56^ mTRAQ,^57^ and iDiLeu^58^ that can introduce mass differences at the precursor level. These tags are often variants of their isobaric version, differing in the number of heavy isotopes. We will introduce the structures of these tags in Section 2.3.

In summary, precursor ion-based quantification methods are realized by comparing peak area in MS1 spectra. Due to the specific *m*/*z* of each precursor ion, these methods offer high quantitative accuracy.^29^ However, due to the complexity of proteomic samples, many coeluting peptides share similar precursor ion masses in MS1 spectra, and the S/N of MS1 peak is often lower than MS2. Thus, quantitation data for low-abundance species are less reliable. Additionally, the use of isotopes introduces multiple peaks for the same peptide, thus, increasing MS1 spectral complexity. This complexity can cause multiple MS/MS triggering for the same peptide, producing redundant MS2 spectra.^30^ Also, to minimize isotopic envelope interference, these methods require a mass difference of >4 Da, limiting the throughput.

### 2.2 Mass Defect-Based Precursor Ion Quantification

Mass defect-based precursor ion quantification is a novel method that addresses the limitations of traditional MS1-based quantification methods, which typically require a mass difference of 4 Da. This approach utilizes the concept of mass defect, which means the neutron-binding energies of different stable isotopes result in small but distinguishable mass differences (commonly at the mDa level). Commonly used isotopes include ^12^C/^13^C (+3.3 mDa), ^1^H/^2^H (+6.3 mDa), ^16^O/^18^O (+4.2 mDa), and ^14^N/^15^N (−3.0 mDa).^59^ These tiny mass differences are indistinguishable in low-resolution but can be resolved using high-resolution instruments like Fourier Transform Ion Cyclotron Resonance (FT-ICR) and the Orbitrap instrument, thereby enabling multiplex quantification without increasing complexity of the MS1 spectrum (Figure 2B).

**Figure 3:**
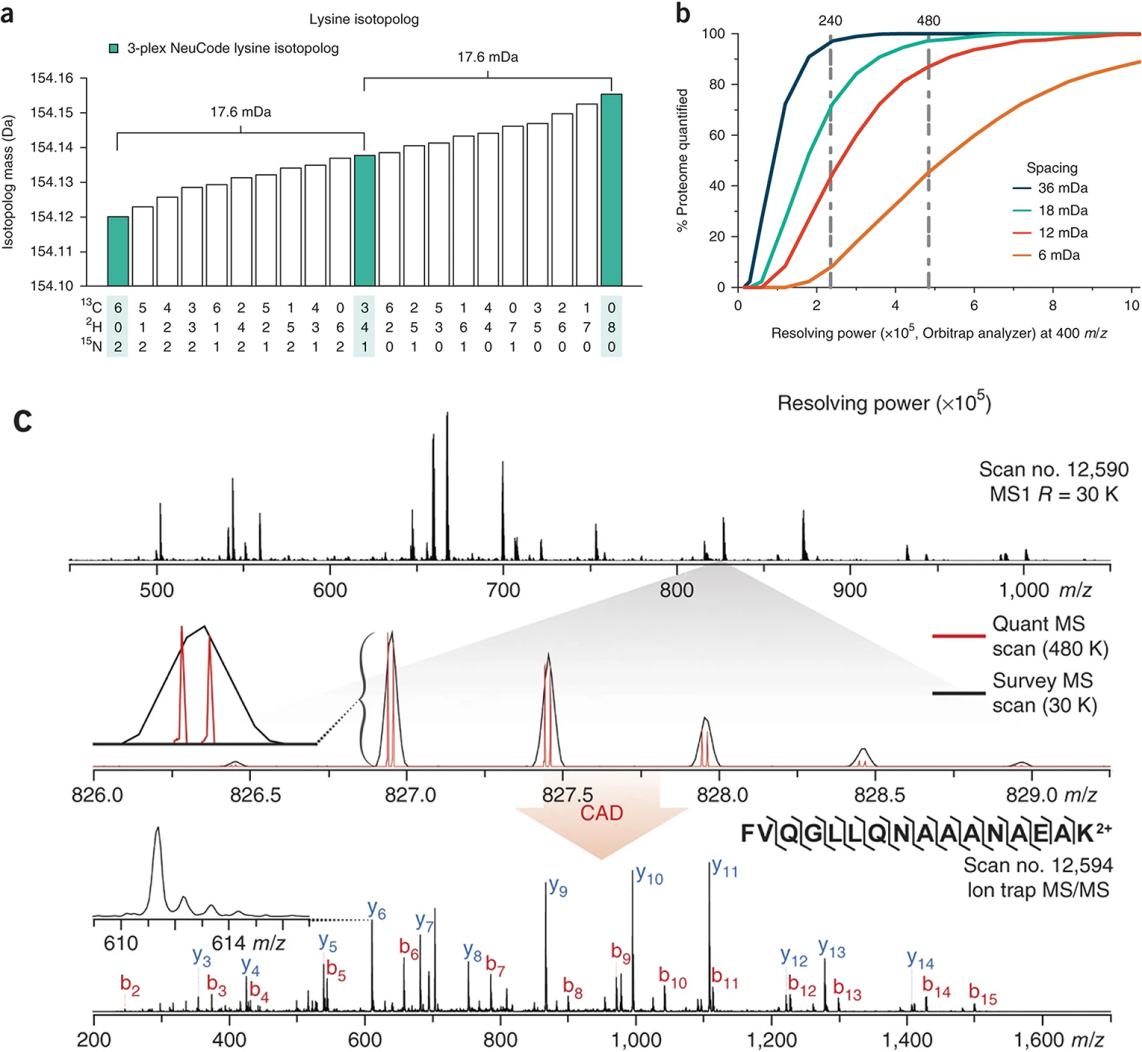
Illustration of NeuCode SILAC Strategy. (a) Theoretical mass of the possible isotopologues for a + 8 Da lysine amino acid. (b) Theoretical calculation depicting the percentage of peptides that are resolved when spaced 6, 12, 18, or 36 mDa for different resolving powers 15 thousand to 1 million. (a,b) Reproduced or adapted with permission from ref (59). Copyright 2013, Springer Nature. (c) MS1 scan collected with 30,000 resolving power of yeast LysC peptides and inset of a selected precursor having *m*/*z* at 827 (black trace). The signal recorded in a subsequent high resolution MS1 scan (480,000 resolving power) is shown in red–only at this high resolution is the quantitative data revealed. Presented below the MS1 scan is an MS/MS spectrum of the neutron encoded SILAC pair. Collisionally activated dissociation (CAD), also known as collision-induced dissociation (CID), was applied to generate MS/MS spectrum. Reproduced or adapted with permission from ref (235). Copyright 2018, Springer Nature.

This mass defect principle was first incorporated into metabolic labeling. The Neutron Encoded (NeuCode) SILAC concept pioneered by Hebert et al.^59^ employs two types of isotope-encoded lysine to culture yeast cells: one with six ^13^C atoms and two ^15^N atoms, and another with eight deuterium atoms. This labeling introduces a 36 mDa mass difference, which is distinguishable at MS resolutions exceeding 200 K (Figure 3). Merrill et al.^31^ expanded this to a 6-plex neutron-encoded SILAC experiment using additional lysine isotopologues, each with a 6 mDa difference, requiring a 960 K resolving power for accurate quantification.

Chemical labeling is another method for introducing mass defects. Hebert et al.^60^ first reported mass defect-based chemical labeling, employing amine-reactive NeuCode tags composed of acetylated arginine- acetylated lysine- glycine. By incorporating six heavy isotopes in various configurations, they created a set of 4-plex labels with each label displaying a 12.6 mDa difference. However, the tags potentially hinder peptide identification rates due to the generation of noninformative tag-related product ions and arginine’s suppression of peptide chain cleavage. Thus, a more compact, less intrusive, mass defect-based tag would be more beneficial for effective peptide identification.

Our group has advanced amine-reactive chemical labels with mass defects. Frost et al.^61^ introduced the Dimethyl Pyrimidinyl Ornithine (DiPyrO) tags for quantitative proteomics. These tags are synthesized by reacting arginine with acetylacetone, yielding dimethyl pyrimidine derivatives. This process effectively reduces the level of peptide fragmentation suppression caused by arginine. Triplex and 6-plex quantifications are achievable at resolutions of 240 and 480 K, respectively, with an 8-plex variant proposed for future higher-resolution applications. Hao et al.^62^ explored duplex mass defect-based *N*,*N*-dimethyl leucine (mdDiLeu) tags for proteomics and amine-containing metabolomics quantification. Zhong et al.^63^ further extended this approach to a 5-plex method by combining isotopic and mass defect labeling. With the compact structure of the mdDiLeu label, each mdDiLeu tag was synthesized efficiently in a single step with readily available materials. However, the incorporation of deuterium atoms into these tags might induce retention time shifts.

Mass defect tagging also stands out for its ability to generate distinct mass signatures, facilitating the selection of diagnostic peaks for accurate peptide identification and quantification. Tagging light and heavy mass defect labels in equal ratios can create distinct peak pairs with tiny mass difference for analytes, which is recognizable by software.^64^ For example, Di et al.^65^ utilized sialic acid chemical labeling to introduce unique mass defect patterns for enhanced detection and quantification of sialylated glycopeptides in complex samples. Similarly, Wang et al.^66^ developed the mNeuCode (methyl-neutron-coding) method for targeted analysis of protein arginine dimethylation, using metabolic labeling to produce diagnostic mass defect peak pairs.

Overall, mass-defect methodology addresses the challenge of precursor interference, outperforming traditional MS1-based quantification methods in sampling depth and multiplexing capacity. However, it requires ultrahigh MS1-level resolutions (typically >200 K at *m*/*z* 200) to differentiate subtle mass differences on peptide precursors, necessitating advanced FT-ICR or Orbitrap instruments and longer cycle times. Multiple isotope incorporation is normally required to generate MS-resolvable mass difference. Thus, the mass defect’s multiplexing potential is limited by the availability of isotopologues.^61^ Also, achieving higher multiplexing will inevitably involve deuterium atoms, which could cause retention time shifts.

### 2.3 Reporter Ion-Based Quantification

Reporter ion-based quantification methods significantly improve throughput compared to precursor ion-based methods through the utilization of isobaric tags.^2,67^ Isobaric tags have identical masses but differ in their isotopic composition. They are normally composed of three parts: a reporter group to generate reporter ions under fragmentation, a mass balancer group to ensure a consistent mass across different channels, and a reactive group that attaches the tag to peptides.

Peptides from different samples will be labeled with different plexes of isobaric tags and mixed for analysis. In MS, labeled peptides will generate only a single peak in MS1 analysis due to the identical chemical composition and mass of isobaric tags. However, during MS/MS fragmentation, the distinct reporter ions are released, allowing for the measurement of peptide abundance in each sample based on the intensity of the corresponding reporter ion. Compared with precursor ion-based quantification, reporter ion-based quantification only relies on the signal intensity of the reporter ions from tandem MS, without the need to construct extracted ion chromatograms (Figure 2C).

This method simplifies the MS1 spectra by generating multiplex labeled peptides as a single composite peak, enabling high-throughput quantification without an increase in spectral complexity. Moreover, by aggregating signals for the same species from all isobaric labeling channels, the MS1 signal is boosted due to the same *m*/*z* of the differently labeled peptides. Thus, the signal of peptide fragment ions in the MS/MS spectrum can also be enhanced, facilitating improved peptide identification and overall proteome coverage.

Over the past decades, various isobaric tag sets have been developed, each with unique chemical structures, as will be detailed. The mass defect concept has also been adopted into the reporter ion group to increase the multiplex capability. As the mass of the reporter ion is much lower than the intact peptide precursors, a substantially lower resolution power (typically >50K at *m*/*z* 200) is sufficient to resolve the reporter ion with several mDa level mass defect, compared with mass defect-based precursor ion quantification methods (typically >200 K at *m*/*z* 200 is required).^68,69^

#### 2.3.1 Commercially Available Reagents: TMT, TMTpro, and iTRAQ

**Figure 4:**
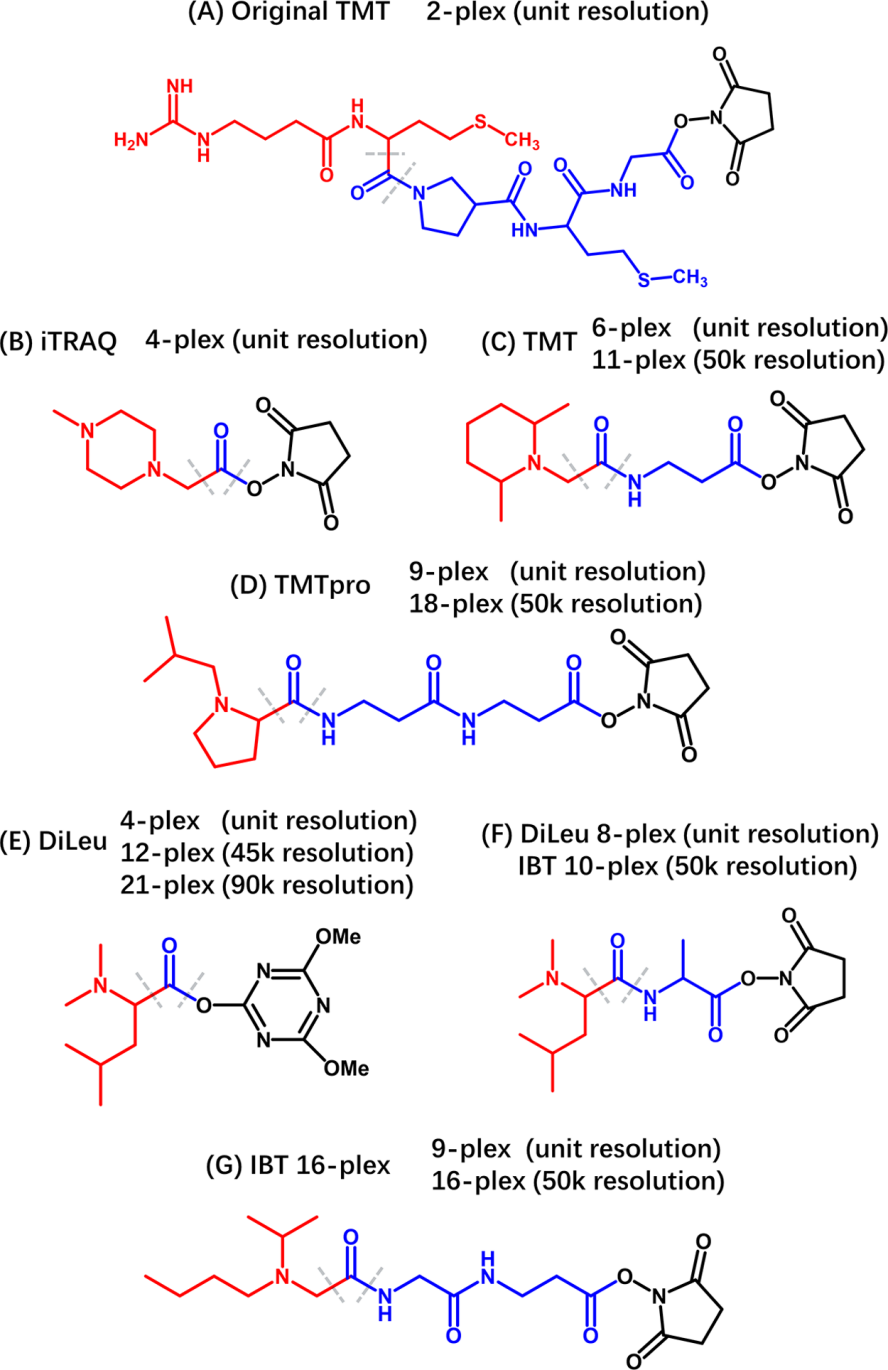
Chemical structures of different isobaric tags applied in quantitative proteomics. The red part of the structure indicates the reporter group; the blue part indicates the mass balancing group; and the black part indicates the amine reactive group. For each tag, the required resolution (at *m*/*z* = 200) to achieve baseline separation of the reporter ions is listed, providing information for the selection and application of these tags based on MS instrument resolutions. The dash lines represent HCD/CID cleavage site. For proteomics-based applications, HCD fragmentation is normally applied to generate reporter ions due to the low mass cutoff (or “one-third rule”) of CID activation for ion trap.^4^

In 2003, Thompson et al.^70^ introduced the Tandem Mass Tag (TMT) concept. The initial design of TMT was a duplex tag system composed of amino acids, integrating an isotopically labeled amino acid tag, a mass normalization amino acid, a cleavage enhancement group (proline), and an *N*-hydroxy succinimide (NHS) ester as an amine-reactive group (Figure 4A). This design facilitated the concurrent acquisition of both peptide backbone and reporter ions through collision-induced dissociation (CID) or higher-energy collisional dissociation (HCD), enabling simultaneously relative quantification and identification in MS/MS spectrum.

Following the first version of duplex TMT, the isobaric tag for absolute and relative quantification (iTRAQ) was introduced with a 4-plex tag design.^71,72^ This system utilized a more compact *N*-methyl piperazine reporter ion group and a carbonyl balance group (Figure 4B). iTRAQ was later expanded to an 8-plex configuration by incorporating additional stable isotopes and enlarging the balance group.^73^ The mass of reporter ions was carefully designed to avoid peak overlap with peptide intrinsic fragmentation ions, such as the phenylalanine immonium ion.

Subsequent advancements led to a refined 6-plex TMT,^74^ featuring a dimethylpiperidine reporter ion and a more streamlined balance group, while preserving the NHS moiety as the reactive group (Figure 4C). Compared with the first-generation of TMT, the newly developed TMT tag was more compact and was optimized for reporter ion formation. Subsequently, this 6-plex TMT was expanded to 11-plex labeling without changing its chemical structure.^68^ This was achieved through the tiny mass difference of 6.3 mDa between the ^13^C and ^15^N isotopes, which could be differentiated at a resolution of 50K or higher (at *m*/*z* 200) for the reporter ions. Due to the availability of isotopologues, this version of TMT is limited to 11-plex while with the potential of reaching 18-plex.^3^

To meet the demand for higher multiplexing capacities, the TMTpro series was recently introduced.^75−77^ This series adopts isobutylproline for reporter ion generation and integrates two β-alanine residues as balance groups (Figure 4D). This novel structure allows for the incorporation of stable isotopes with less synthesis effort and lower cost. By incorporating nine stable heavy isotopes and utilizing the mass defect between ^13^C and ^15^N isotopes, up to 18-plex TMTpro labeling can be achieved. Compared to TMT 11-plex reagent, TMTpro exhibits higher hydrophobicity and requires lower fragmentation energy in MS/MS. Comparative studies have shown similar performance in peptide identification numbers and quantification accuracies between TMTpro 16-plex and TMT 11-plex reagents.^77^

Among the commercially available isobaric tags, the TMT/TMTpro series has gained popularity, predominantly for two reasons: the high multiplexing capacity and the integration of TMT-centric templates by Thermo Fisher Scientific in the latest Orbitrap instruments and data processing software. This integration significantly streamlines the workflow, positioning TMT reagents as an increasingly accessible choice for researchers.

#### 2.3.2 Cost-Effective Alternatives: DiLeu and IBT

While isobaric labeling methods like TMT and iTRAQ have gained popularity in quantitative proteomics, their widespread application has been hindered by the high cost of reagents, primarily due to the intricate and multistep synthesis processes.^69^ Consequently, there is a demand for cost-effective isobaric tags.

*N*,*N*-Dimethyl leucine (DiLeu), developed in our laboratory, emerges as an efficient alternative. It was inspired by the generation of intense a1 ions post peptide dimethylation with leucine at the N-termini.^54,78−80^ Initially conceptualized as a 4-plex tag by Xiang et al.,^80^ DiLeu boasts a compact structure, comprising a reporter group, a carbonyl balance group, and an amine-reactive triazine ester group (Figure 4E). DiLeu’s advantages include the cost-effectiveness and high synthesis yield for various channels while maintaining high labeling efficiency and accurate protein quantitation. In comparison to TMT-labeled peptides, DiLeu tags also demonstrate improved peptide backbone cleavage and more intense reporter ion signals, facilitating the detection and quantitation of low-abundance peptide species.

Later, the multiplexing capacity of DiLeu was expanded from a 4-plex to a 12-plex system, utilizing mass defects from ^15^N, ^13^C, and D isotopes.^69^ This expansion requires a resolution above 45K at *m*/*z* = 200 to achieve baseline separation of 12 reporter ions with a minimum spacing of 5.8mDa. Notably, seven of these channels necessitate only a single-step synthesis, while the remaining five channels require one additional ^18^O exchange step, enhancing the multiplex capability while preserving the synthesis-effectiveness. More recently, DiLeu’s multiplexing capability was escalated to a 21-plex through stepwise monomethylation, with a minimal spacing of 3 mDa between neighboring channels, demanding higher resolution (90K at *m*/*z* 200) for baseline resolve on FT-MS instruments.^81^

In its chemical composition, DiLeu employs 4-(4,6-dimethoxy-1,3,5-triazin-2-yl)-4-methylmorpholinium (DMTMM) triazine ester as the amine reactive group, necessitating an activation process prior to labeling. Stored in its carboxylic acid form, DiLeu has a longer shelf life and is free of hydrolysis degradation problem compared with NHS esters applied in TMT and iTRAQ.^82^ Both triazine ester and NHS ester can achieve complete labeling of peptide amine group within the labeling process. However, the DiLeu labeling process mandates additional purification step (like strong cation exchange) to remove excess activation reagent.^83^

The compact structure of DiLeu requires the incorporation of deuterium atoms to extend the multiplexing capability. By placing deuterium atoms in the hydrophilic dimethylamine group, the deuterium shift effect is minimized.^30,52,69,81^ It is noteworthy that 12-plex and 21-plex DiLeu do not have completely identical precursor masses but exhibit slight differences at the mDa level, as also observed with 18-plex TMTpro. This results in uncertain mass increments in MS1 for labeled peptides (normally around the average mass of all channels). Despite these variations, these labeling methods are still classified under isobaric labeling, as the subtle differences are not resolved in MS1 under low resolution to differentiate labeling channels. Also, these variations will not adversely affect data acquisition and protein identification workflows.^69,76,81^

An alternative 8-plex version of DiLeu-based tags, not dependent on mass defect, increases multiplexing capacity by integrating larger balancer groups and more stable isotopes (Figure 4F).^84^ The reporter ions of these isobaric tags are set 1 Da apart, permitting application on low-resolution instruments. However, a more significant retention time shift happens between channels due to deuterium placement in the balance group.

Apart from DiLeu, the Isobaric Tag (IBT) serves as another cost-effective alternative utilizing amino acids as building blocks. IBT accomplishes 10-plex labeling exclusively using ^13^C and ^15^N isotopes, thereby minimizing deuterium-induced retention time shifts.^85^ The recently unveiled IBT-16plex introduces isopropylated–isobutylated glycine as the reporter group, complemented by glycine and β-alanine in the balance group (Figure 4G).^86^ This configuration intentionally reduces reporter ion intensities to amplify b/y-ion production, thus, enhancing peptide identification. Comparative studies have revealed that IBT-16 plex substantially outperforms TMTpro-16plex in peptide and protein identification numbers in HeLa cell samples.^86^

#### 2.3.3 Summary

In summary, reporter ion-based quantification methods enable increased multiplexing capability while avoiding the problems of spectrum complexity in precursor-based quantification techniques. Although these methods demonstrate excellent throughput, they increasingly suffer from ratio distortion caused by the coisolation of precursor ions and subsequent cofragmentation of peptides. This issue adversely affects the accuracy and precision of these methods.^2,3,25,29,83,87^ We will discuss this challenge in section 3 and detail various methods developed to address this challenge.

### 2.4 Hybrid Quantification Methods

Recently, the pursuit of improved multiplexing capabilities and quantitative accuracy has led to the development of hybrid (or hyperplex) labeling methods. The fundamental concept is utilizing mass differences at the precursor level to enable the simultaneous analysis of multiple sets of isobaric labeled samples. Hybrid quantification approaches are typically achieved through two kinds of methods:

#### 2.4.1 Combining Isotopic Labeling and Isobaric Labeling

**Figure 5:**
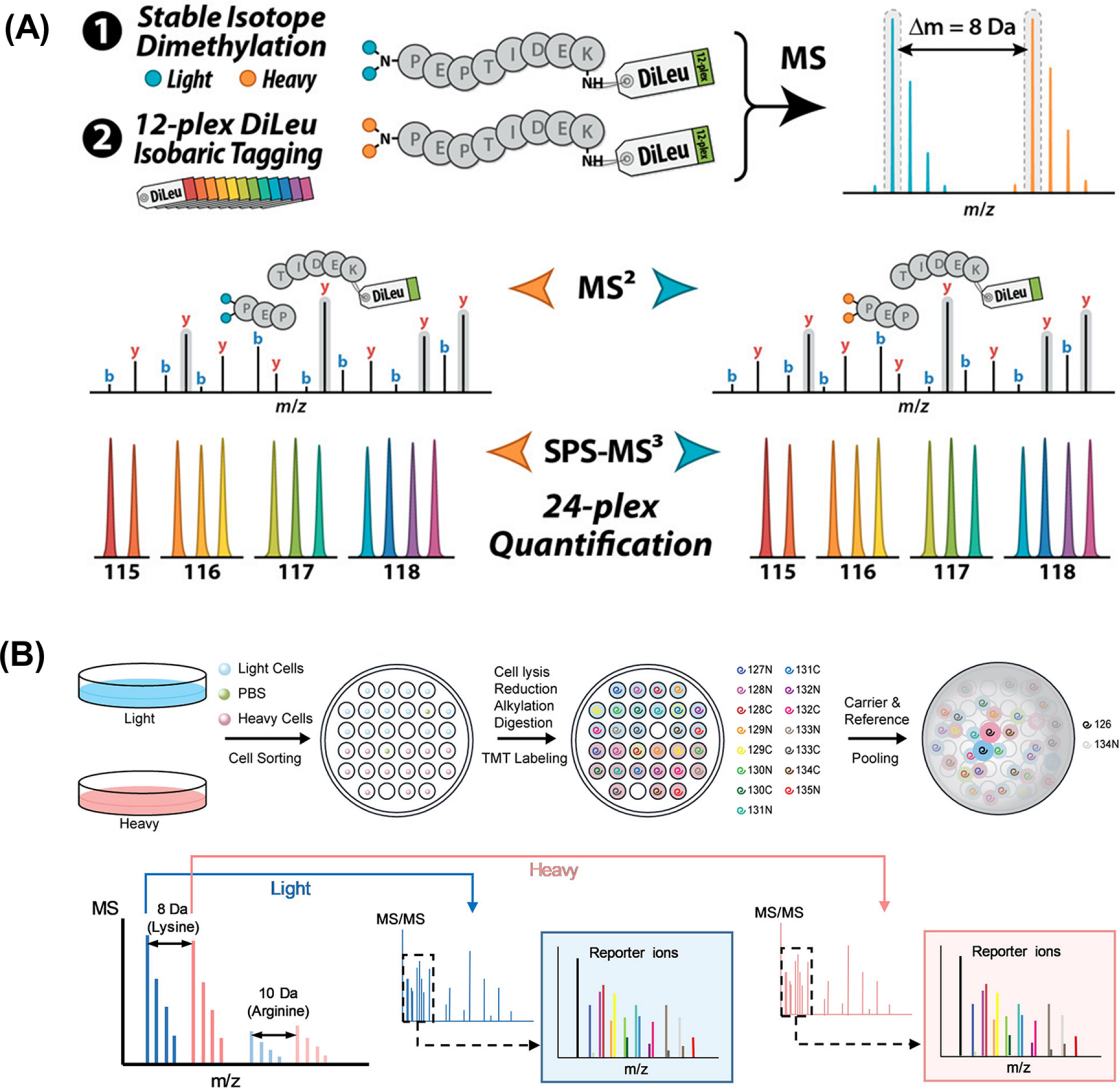
(A) DiLeu cPILOT experimental workflow. Digested samples undergo stable isotope dimethyl labeling of peptide N-termini with either light or heavy dimethyl groups at low pH followed by labeling of lysine residues with 12-plex DiLeu isobaric tags at high pH. Pooled samples are analyzed by LC-MS/MS using CID MS2 acquisition of peak pairs for peptide sequence identification and HCD SPS-MS3 acquisition for accurate 24-plex quantification via DiLeu reporter ions. Reproduced or adapted with permission from ref (88). Copyright 2018, American Chemical Society. (B) Hyper plex single cell proteomics workflow combining SILAC and TMTpro labeling. First, the cells were cultured separately in regular media (light) or media containing ^13^C_6_^15^N_2_l-lysine and ^13^C_6_^15^N_4_l-arginine (heavy). After cell lysis, reduction, alkylation and digestion, each single cell sample was labeled with a different TMT reagent. The carrier and reference channels were added after quenching, heavy and light precursors were identified in MS1, selected for fragmentation, and analyzed by tandem MS. Reporter ions are produced for relative quantification. Reproduced or adapted with permission from ref (42). Copyright 2023, American Chemical Society.

For example, the combined precursor isotopic labeling and isobaric tagging (cPILOT) method utilizes p*K*_a_ differences between free amines on peptides.^88−90^ It selectively dimethylates the N-terminus amine group to introduce a mass difference, followed by lysine residue tagging with DiLeu or TMT isobaric reagents (Figure 5A). cPILOT methods can expand the multiplexing capability of original isobaric tags by two or three times. Another work achieved tandem labeling in one pot reaction combining mTRAQ and TMTpro,^91^ which minimizes the sample loss and reduces sample preparation times. However, these methods limited the protease to LysC to create double amine groups on peptide termini.^88^ TAG-TMTpro method was reported as a more universal method.^92,93^ It introduces mass differences by labeling *tert*-butyloxycarbonyl (Boc) protected alanine or glycine amino acid residues into peptide amine groups, followed by the deprotection and isobaric labeling on newly introduced amine groups in these amino acid residues. This method allowed for up to 54-plex quantification when combined with 18-plex TMTpro and up to 102-plex when combined with 18-plex TMTpro and 16-plex IBT isobaric tagging.^92,93^

Other strategies include combining SILAC with isobaric labeling. Recently, 2-plex SILAC combined with TMTpro has been applied to enhance the throughput of single-cell proteomics, enabling the analysis of up to 28 single cells with carrier and control channels in a single LC-MS run (Figure 5B).^42^

#### 2.4.2 Labeling Samples with Multiple Sets of Isobaric Tags

For instance, by mixing 11-plex TMT, 18-plex TMTpro, and 16-plex IBT labeled samples, up to 45-plex quantification can be achieved.^94^ Other methods have also been reported recently with different tag combinations.^95,96^ For further insights into these methods, we recommend reading the review article by Bowser et al.^97^

One significant advantage of hybrid labeling methods is the substantial increase in throughput. This approach can save considerable instrument time and reduce sample preparation variability.^97^ However, these strategies inherit issues from both precursor and reporter ion-based quantification methods, including increased spectral complexity at the precursor level, redundant MS2 spectra, and reporter ion ratio distortion problem.^3^ Also, when combining multiple sets of isobaric tagging, missing values of peptide identification may happen between different isobaric groups due to the ionization efficiency variation of isobaric tags, which will influence the reproducibility of proteins identified between groups.^93^

## 3 Challenges in Reporter Ion-Based Quantitative Proteomics and Solutions

While isobaric labeling methods have become one of the most employed approaches for multiplex quantitative proteomics, they present notable challenges that may impact experimental outcomes. A significant problem is ratio distortion (or compression) due to coisolation and cofragmentation, which can severely affect the accuracy and precision of quantitative results.^2−4,83,87^

**Figure 6:**
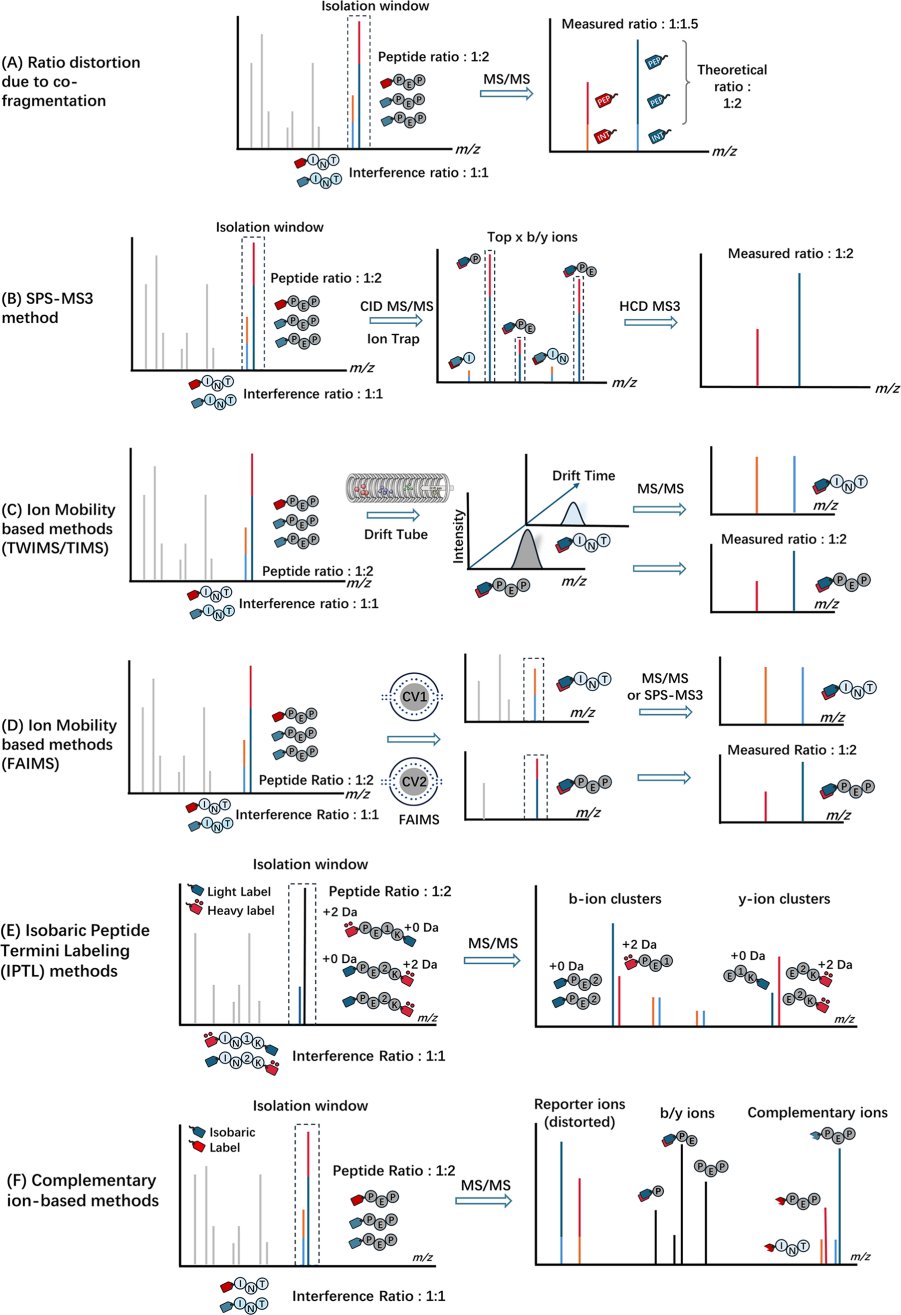
(A) Illustration of ratio distortion problem in reporter-ion based quantification. PEP represents peptides from samples and INT represents coeluted interference peptides. Peptides sharing similar *m*/*z* values and retention times are coisolated and cofragmented in MS/MS, leading to quantification inaccuracies due to interference. This is represented by the merging of reporter ions in the MS2 spectrum, where the measured ratio is compressed compared with the theoretical ratio. (B) Illustration of SPS-MS3 method for reducing ratio distortion. The highest abundance peaks of ion trap MS/MS, which typically are b and y ions from the peptide of interest, are simultaneously isolated and further fragmented for an MS3 spectrum. Reporter ions in the MS3 spectrum are used for quantification. (C) and (D) Illustration of ion-mobility based strategies (TWIMS/TIMS and FAIMS, respectively) that provide extra gas-phase separation to separate coeluting peptide ions, utilizing additional separation dimensions such as drift tube (in TWIMS/TIMS) or compensation voltage (CV) changes (in FAIMS) to mitigate ratio distortion effects. (E) Schematic overview of the isobaric peptide termini labeling (IPTL) approach, where the red dot symbol above the heavy label symbol represents incorporated heavy isotope. PE1K represents peptides from sample 1, and IN1K represents interference peptides from the same sample. Similarly, PE2K and IN2K correspond to sample 2. The peptide-specific backbone ion pairs are employed for quantification. (F) Schematic overview of complementary ion-based methods, which leverages the peptide-specific complementary ion for quantification. These complementary ions provide accurate quantitative ratios, in contrast to reporter ions, which can be distorted due to cofragmentation interference.

In data-dependent acquisition (DDA)-based methods, a predefined isolation window selects ions for MS/MS fragmentation. Ideally, this isolation window isolates a single peptide precursor. However, due to the technological limitations of ion filters, the smallest achievable isolation window with ion filters, like quadrupole, is approximately 0.4 Th.^98^ Any coeluting peptide ions within the mass isolation window will be coisolated and fragmented. The reporter ion ratios obtained in the MS/MS spectra will be distorted, as the reporter ions from contaminating precursors are indistinguishable from the target analytes (Figure 6A).

Typically, the issue of ratio distortion can be evaluated using a dual proteomics model,^99,100^ which involves labeling samples from different origins in various ratios (e.g., 5:2:1 for human-derived samples as study channel and 1:1:1 for yeast as interference) then mixing them for MS analysis. This approach allows for the assessment of the extent of ratio compression between different analytical methods by comparing the experimental ratios and theoretical ratios. More recently, an isobaric tag-based Triple Knockout (TKO) quality control platform has been developed and commercialized by Gygi and co-workers,^101,102^ providing a new standard for optimizing MS data collection parameters to reduce ratio distortion.

Various strategies have been developed to mitigate ratio distortion in isobaric-labeling-based proteomics, which can be classified into three categories: (1) Modification of instrument acquisition settings; (2) application of an extra level of separation (Pre LC-fractionation; MS3; and Ion mobility); and (3) use of peptide-specific ions for quantification (Peptide fragment ion; Complementary ion). A comparative analysis of these methods is summarized in Table 1. This section aims to guide readers through the principles of each approach, assisting in the selection of the most suitable method for specific research needs.

**Table 1:** Comparative Analysis of Methods for Mitigating Ratio-Compression Problem

methods	effect on mitigating ratio distortion^a^	special instrument required	influence on quantified peptides ID number	influence on analysis time and throughput	influence on data processing	other comments
modification of acquisition settings	+	no	N/A	N/A	N/A	easy to apply
sample fractionation	++	HPLC with fraction collector	increase	increase total analysis time	increase database search time	can be combined with other methods
MS3-based methods	+++	Orbitrap Tribrid MS	decrease due to extra duty cycle	N/A	N/A	gold standards for complex samples
ion-mobility based methods	+++	MS coupled with ion mobility spectrometry or FAIMS interface	increase	TIMS/TWIMS: ToF analyzers limit reporter ion to unit mass difference	need software compatible with IMS data interpretation	In-FAIMS fragmentation can cause false identification for glycopeptides samples
peptide fragment ion-based methods	++++	no	decrease	limited plex compared to normal isobaric labeling	need special software for b/y ion cluster identification/extraction	b/y ion clusters make MS/MS spectrum complex
complementary ion-based methods	++++	no	decrease (depending on the efficiency of complementary ion generation)	limited plex compared to normal isobaric labeling; reporter ion is limited to unit mass difference	need special software for complementary ion extraction and deconvolution	normally combined with narrow isolation windows to isolate monoisotopic peak

a Symbols: +, partially effective; ++, good; +++, very good; ++++, excellent.

### 3.1 Modification of Instrument Acquisition Settings

Narrowing the precursor isolation window by reducing the MS/MS isolation window size can effectively lower the number of interfering ions within the window, thereby minimizing coisolation.^103^ Thermo Fisher Scientific recommends setting a 0.7 Th narrow isolation window for analyzing TMT labeled samples, as recommended in their TMT application notes.^104,105^

Additionally, triggering MS/MS at the LC peak apex, known as delayed fragmentation, can significantly decrease cofragmentation by a factor of 2.^103^ These strategies are advantageous, because they generally do not necessitate extra sample processing steps and are straightforward to apply. However, it is important to note that these methods may only partially mitigate ratio distortion.^100^

### 3.2 Pre-LC-MS Sample Fractionation

Prefraction methods such as high-pH reversed-phase LC,^106−108^ strong cation exchange (SCX),^109^ and Hydrophilic Interaction Liquid Chromatography (HILIC)^110,111^ fractionation can effectively decrease sample complexity, thus reducing ratio suppression. However, this approach increases the total analysis time due to the additional fractionation of samples.

### 3.3 MS3-Based Methods

Ting et al.^112^ pioneered the MS3 approach to tackle the challenge of ratio distortion utilizing Orbitrap tribrid mass spectrometers. This technique involved an additional fragmentation step: the most intense peptide fragment ion was further fragmented in MS3 to produce reporter ions. This extra level of separation significantly reduced interference ions, as coisolated peptides are less likely to generate intense peptide fragments ions. Building on this, the MultiNotch MS3 (or Synchronous Precursor Selection-MS3, SPS-MS3) method was introduced to address the sensitivity issues of the original MS3 technique.^99^ It allowed for the systematic coisolation of multiple peptide fragment ions in the ion trap, significantly improving the method’s sensitivity (Figure 6B). Now SPS-MS3 has become the gold standard for analyzing isobaric labeled complex samples.^113^

The latest advancement in this method is the integration of real-time database searching (RTS) with the newest generation of tribrid Orbitrap instruments.^114,115^ RTS adaptively triggers MS3 scans only when reliable peptide identifications occur in the ion trap, significantly increasing the scan efficiency and enhancing the depth of proteome analysis.

### 3.4 Ion-Mobility Based Methods

Ion mobility spectrometry (IMS) offers a novel approach to reduce coisolation. With its orthogonality to LC separation, IMS can provide complementary gas-phase separation to separate coeluting peptide ions and background ions. The bonus of IMS separation is it can enhance the depth of proteomic analysis without increasing total analysis time.^116^

Different IMS platforms have been applied in quantitative proteomics analysis (Figure 6C). Shliaha et al. combined traveling wave ion mobility separation (TWIMS) with narrowed quadrupole isolation to reduce precursor coisolation.^116^ Also, Ogata et al. used trapped ion mobility spectrometry (TIMS) to reduce ratio compression, achieving quantification results comparable with the SPS-MS3 method without compromising instrument sensitivity or speed.^117^

High-field asymmetric waveform ion mobility spectrometry (FAIMS) is the only commercial IMS compatible with Orbitrap mass analyzers and existing SPS-MS3 methods (Figure 6D).^118^ FAIMS has been shown to significantly enhance the dynamic range and accuracy of quantitative measurements without sacrificing protein identifications.^119−121^ FAIMS-SPS-MS3 method was reported to have the highest quantitative accuracy followed by SPS-MS3, FAIMS-HRMS2, and HRMS2.^119,122^ Recently, Fang et al. highlighted the utility of FAIMS in improving the accuracy of N-glycopeptide quantification, showcasing its versatility in post-translational modifications (PTM) analysis.^122^ However, Rangel-Angarita et al. also reported that in-FAIMS fragmentation can lead to false positive detection of glycopeptides, especially when high compensation voltage (CV) values was applied.^123^

### 3.5 Peptide Fragment Ion-Based Methods

Ratio distortion in reporter ion-based methods stems from nondistinguishable reporter ions, leading to inaccuracies when peptide ions are cofragmented. Peptide backbone fragment ion-based methods, like Isobaric Peptide Termini Labeling (IPTL) introduced by Koehler et al.,^124−126^ address this problem by applying peptide-specific fragment ions, ensuring accurate quantification even for cofragmented peptides. IPTL sequentially labels the Lys and N-terminal amine groups with stable isotopes, producing the same peptide mass in MS1 but distinct peptide backbone ions upon fragmentation (Figure 6E). Then precise quantification can be achieved by comparing the intensity of peptide backbone ions. IPTL can increase the confidence of quantification as each peptide fragment ion will carry quantification information.

Despite its advantages, IPTL has limitations of restricted multiplexing capacity compared with common reporter-ion based methods. Recent advancements, like multiplex pseudoisobaric dimethyl labeling (m-pIDL),^127^ have expanded multiplexing capabilities by using large isolation window to isolate pseudoisobaric precursors. However, complex isotopic envelopes created in MS/MS still require advanced data processing. Software tools like IsobariQ^128^ and ITMSQ^129^ were also developed to enhance the identification rates of b/y ion clusters and assist the deconvolutions of quantification results.

Further innovations were developed to reduce the spectral complexity to increase the peptide identification rate. Zhou et al.^130^ used the concept of mass defect to decrease the complexity with requiring high-resolution instruments. Tian et al.^131^ recently developed isobaric acetyl-isoleucine-proline-glycine (Ac-IPG) tags to reduce spectrum complexity by allowing b-ions to remain identical while y-ions isotopically labeled.

Other methods include specifically applying peptide a1 ions from the neutral loss of b1 ions for quantification. Zhang et al.^132^ recently established the 8-plex a1 ion-based proteome quantitation (APQ) method, utilizing isotopic labeled a1 ions for quantification. They further developed the deep-APQ method by sequentially acquiring high and low mass ranges for identification and quantification, offering deeper peptide coverage and higher quantitative accuracy.^133^

### 3.6 Complementary Ion-Based Methods

**Figure 7:**
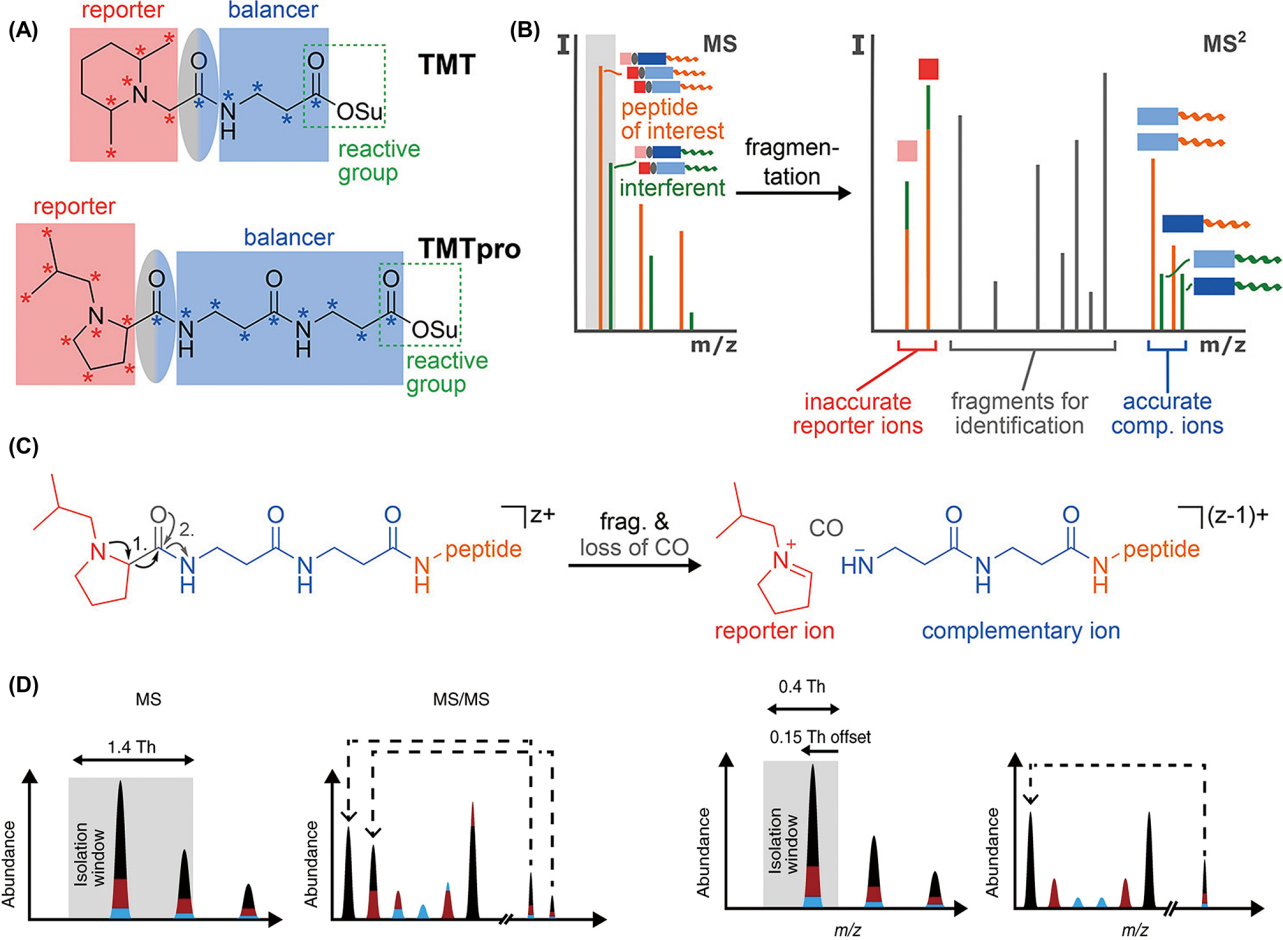
Complementary ion-based quantification methods. (A) TMT- and TMTpro-tags comprise a reporter region (red), a balancer region (blue), and an amine-reactive NHS-ester moiety (green rectangle). The carboxyl group loss as CO during fragmentation is part of the balancer and highlighted in an ellipse (gray-blue). (B) When analyzing complex samples via shotgun proteomics, if MS2 reporter ions are used for quantification, the interfering peptides lead to a distortion of the measured ratios, as the source of reporter ions cannot be distinguished. However, because the masses of complementary ions are peptide-dependent and include the heavy isotope labels of the balancer region, they can be used for interference-free MS2 quantification. (C) During fragmentation of a TMTpro-modified peptide, the positively charged reporter ion is separated from the ion and a neutral CO molecule is lost. This leads to an ion where the balancer part is still attached to the peptide. Because the balancer region encodes the complementary heavy isotope labels of the reporter ion, the balancer-peptide conjugate is called the complementary ion. The charge state of the precursor ion is reduced by one. (A–C) Reproduced or adapted with permission from ref (137). Copyright 2021, American Chemical Society. (D) Left, coisolation of the natural isotope cluster in a standard isolation window centered on the precursor ion convolutes the relative abundance of peptide-coupled reporter ions. Right, an asymmetric narrow isolation window that reduce the signal from adjacent isotope peaks and enables direct quantification of complementary ions. Reproduced or adapted with permission from ref (138). Copyright 2018, Springer Nature.

Another strategy is based on complement reporter ion (or peptide-coupled reporter ion) clusters. This method leverages the unique fragments produced due to the loss of isobaric tag reporter ion and CO neutral loss (i.e., the intact peptide ions with the balancer group attached) (Figures 6F and 7B,C).^134−136^ As those ions are specific to each precursor peptides and distinguishable in MS/MS, this strategy ensures accurate quantification even when peptides coisolate.^3^ A significant advantage of this approach over IPTL methods is that it generates the same b/y ions across different labels, thus not complicating database searches. Also, this approach does not necessitate higher-order MS scans, making it more accessible for laboratories without the latest MS equipment.^137^

However, the method also has its challenges. First, the complexity of the isotopic envelope of complementary ions needs additional data processing and deconvolution.^135^ For peptide ions, when a standard isolation window is used, both monoisotopic peak and ^13^C isotopic peak will be coisolated and fragmented. This coisolation leads to 1 Da mass offset, resulting in peak overlap. This overlap complicates complement ion clusters, because the mass difference between each plex is also 1 Da (Figure 7D). Consequently, a deconvolution process is required to reveal the actual ratio. Recent works applied narrow (<0.5 Da) and/or asymmetric isolation window to specifically isolate the monoisotopic peak, thus reducing the need for deconvolution process and increasing the quantification accuracy (Figure 7D).^113,136−138^

Second, the throughput of this method remains limited. Due to the present limitations in the instrument resolving power for large peptide-coupled ions, complementary ions require a 1 Da mass difference. Till now, the highest plex of complementary ion quantification achieved is 8-plex TMTproC reported by Wühr and co-workers (Figure 7).^137^ They also proposed a transient super-resolution technique on the Orbitrap platform as a possible future solution to this problem.^139^

Third, commercial TMT tags suffer from low formation efficiency of complementary ions.^140^ Recent developments, such as the SulfOxide (SO)-tag^141^ and EASI tag,^138^ utilize a sulfoxide-based tag for increased cleavage efficiency in MS/MS, thus enhancing the formation efficiency and yield of peptide-coupled reporter ions. Our group has recently introduced the 7-plex dimethylated leucine complementary ion (DiLeuC) tags.^113^ Compared with commercial TMT or TMTpro tags, DiLeuC tags demonstrate better complementary ion generation efficiency and are more cost-effective. Also, accurate proteome quantification at the single-cell level was achieved, indicating a promising future for this strategy.

## 4 Applications of Labeling Strategies in Quantitative Proteomics

### 4.1 Cancer Proteomics

Cancer cells exhibit abnormal protein expression patterns compared to normal cells.^142^ Proteomics techniques, such as MS and two-dimensional gel electrophoresis (2-DE),^143,144^ are used to analyze these protein expressions to identify cancer-specific proteins (biomarkers) and explore the molecular mechanisms of tumor formation and development.^145,146^ Post-translational modifications of proteins, such as phosphorylation, glycosylation, and ubiquitination, play a significant role in cancer progression.^147^ Proteomics research focuses on these modifications to understand how they alter protein functions and signaling pathways in cancer cells, leading to uncontrolled growth and metastasis of cancer cells.^147^

An important aspect of cancer proteomics is the identification of biomarkers. These biomarkers are proteins whose expression levels significantly change under carcinogenic conditions and can be used for early detection, prognosis, predicting treatment response, and monitoring disease progression.^145,146^Table 2 presents representative cancer biomarkers identified using label-based strategies for protein quantitation.^148−158^ For example, Gao et al. employed TMT technology to analyze hepatocellular carcinoma (HCC) associated with hepatitis B virus.^148^ According to their phosphoproteomics methods, the phosphorylation of ALDOA was found to enhance glycolysis and proliferation in HCC cells with the CTNNB1 mutation, while PYCR2 and ADH1A were related to the metabolic reprogramming in HCC. These findings help researchers better understand and design effective treatment strategies for HCC.

**Table 2:** Representative Cancer Biomarkers Identified Using Labeling Proteomics Approaches

cancer type	sample type	labeling strategy	potential biomarkers	feature of biomarker	author and reference
liver	clinical tissue	TMT	pyrroline-5-carboxylate reductase 2 (PYCR2), alcohol dehydrogenase 1A (ADH1A), phospho-aldolase A (ALDOA)	involved in HCC metabolic reprogramming and increased glycolysis and cell proliferation	Gao et al.^148^
prostate	LAPC4	SILAC	α(1,6)-fucosyltransferase (FUT8)	increased oncogenic activity and metastasis	Clark et al.^149^
	clinical serum	iTRAQ	CD59, haptoglobin, tetranectin	correlated with bone metastasis	Yan et al.^150^
pancreas	clinical serum	iTRAQ	apolipoprotein A-1 (APOA1), transferrin (TF)	correlated with the degree of histological differentiation	Lin et al.^151^
	clinical tissue	TMT	melanocyte inducing transcription factor (MITF), transcription factor binding to IGHM enhancer 3 (TFE3), transcription factor EB (TFEB)	increased activation of anabolic pathways, autophagy, and lysosomal catabolism	Perera et al.^152^
breast	clinical tissue	TMT	fatty acid-binding protein-7 (FABP7)	increased progression and metastasis	Asleh et al.^153^
	MCF7, MDA-MB-231	DiLeu	methylated pyruvate kinase M2 (PKM2)	increased cell proliferation, migration, and metastasis	Liu et al.^154^
ovarian	OV-90	SILAC	calcium-activated chloride channel 1 (CLCA1)	increased cell aggregation	Musrap et al.^155^
	clinical plasma	TMT	fibrinogen alpha chain (FGA), gelsolin (GSN)	correlated with tumorigenesis and metastasis	Zhang et al.^156^
lung	A549	TMT	threonine tyrosine kinase (TTK)	increased tumorigenesis	Chen et al.^157^
	CL1–5	SILAC	karyopherin alpha 2 (KPNA2)	increased cell migration	Wang et al.^158^

### 4.2 Neuroproteomics

Neuroproteomics is a specialized field within proteomics, focusing on the study of the proteome in the nervous system. This field involves comprehensive analysis of proteins in brain tissue, cerebrospinal fluid, and other neural components.^159^ By comparing the proteomic characteristics of healthy and diseased neural tissues, researchers can identify abnormal protein expressions and modifications, thereby revealing the molecular mechanisms of neurological disorders such as Alzheimer’s disease,^160,161^ Parkinson’s disease,^162^ schizophrenia,^163,164^ and multiple sclerosis.^165,166^

Isotope labeling techniques are widely used in neuroproteomics to address various biological questions. These techniques are primarily used to characterize the proteomes of neurological disorders, drug responses, or different regions of the brain.^167,168^ For instance, isobaric labels such as DiLeu, iTRAQ, and TMT have been utilized to study dynamic protein changes in children with B-cell acute lymphoblastic leukemia during chemotherapy, investigate the proteomics of serum in Parkinson’s patients, and analyze circuit-specific proteomes and phosphoproteomes in the corticostriatal system of the mouse brain during development.^169−171^ Additionally, numerous reports have focused on the studies of protein expression in the thalamus and cerebrospinal fluid of schizophrenia patients,^164^ investigation of variations in protein expression levels in human brains after severe traumatic brain injury,^172^ and even exploring the mechanisms of memory formation in the hippocampus.^164,172,173^ Overall, neuroproteomics provides deep insights into the complexity of the nervous system and its related diseases by revealing potential molecular linkages through the comparative quantification of proteome alterations.

### 4.3 Post-translational Modifications (PTMs)

Post-translational modifications refer to a series of chemical and biological changes after proteins are translated, including phosphorylation, glycosylation, acetylation, citrullination, and more.^174^ These modifications can significantly alter the physical and chemical properties of proteins, thereby affecting their function and role within cells and biological systems.^175^ PTMs play a critical role in a variety of cellular processes, such as signal transduction, DNA repair,^176^ and cell cycle control.^177^ Understanding PTMs is crucial for comprehensive proteomic analysis, as aberrant PTMs are often linked to pathogenesis, particularly in studying disease mechanisms, biomarker identification, and drug target discovery.^178,179^

#### 4.3.1 Phosphorylation

Phosphorylation is the process of adding a phosphate group to serine, threonine, or tyrosine residues in proteins. This modification significantly alters the charge and conformation of the protein and is one of the most common PTMs.^180^ Phosphorylation plays a crucial role in regulating protein activity, signaling pathways, and various cellular processes, such as cell division and metabolism.^181^ In proteomics research, MS is the primary method for identifying and quantifying phosphorylation, and tandem mass spectrometry can precisely determine the specific sites of phosphorylation on peptides.^182^

The stable isotope labeling of amino acids in cell culture (SILAC) technique, using [γ-^18^O_4_] ATP labeling, is particularly used for specifically labeling phosphorylation sites. An additional advantage of this technique is the ability to identify and quantify multiple phosphorylation sites simultaneously through mass spectrometry.^183^ Molden et al. demonstrated that SILAC has been used to label and reliably identify over 1,000 nuclear phosphorylation sites in a single experiment, measuring phosphorylation levels of nuclear proteins in different cell cycle states (asynchronous, G1/S, and M phase synchronized), and further identifying the most active phosphorylation sites under these conditions.^184^

Isobaric labeling techniques, such as iTRAQ, TMT, and DiLeu, are also widely used in proteomics to study phosphorylation. These techniques can accurately quantify phosphorylation changes in multiple samples in a single experiment, providing important insights into cellular mechanisms and disease pathology.^185^ Given the low abundance of phosphorylated peptides, workflows in phosphorylation studies often include enrichment techniques such as IMAC or TiO_2_ to simplify samples and improve the detection rate of phosphorylated species.^186^ Jiang et al. successfully identified 12,465 phosphorylated peptides and quantified 10,436 peptides in 10 samples treated with insulin or insulin-like growth factor 1 (IGF-1) in the lung cancer A549 cell line.^187^ This comparative analysis provided detailed information, helping to outline different signaling pathways including mTOR (mechanistic target of Rapamycin), epidermal growth factor receptor (EGFR), and insulin signaling pathways.

#### 4.3.2 Glycosylation

Glycosylation, a key process in protein PTM, involves the addition of carbohydrate structures to proteins. This modification significantly affects protein structure and function, playing an important role in numerous biological processes and diseases.^188,189^ Glycosylation is primarily divided into two types: N-linked glycosylation, where sugar chains are attached to the nitrogen atom of asparagine residues, and O-linked glycosylation, where sugar molecules are attached to the oxygen atom of serine or threonine residues.^190,191^

**Figure 8:**
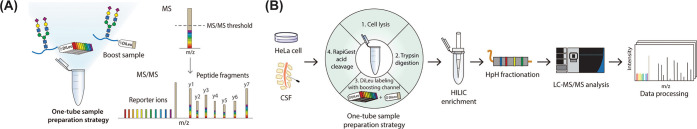
Boost-DiLeu: enhanced isobaric *N*,*N*-dimethyl leucine (DiLeu) tagging strategy. (A) Illustration of a one-tube sample preparation strategy to amplify the signal with isobaric tags for quantitative glycoproteomic analysis. The boosting channel significantly increases the low-abundance glycopeptides. (B) The boost-DiLeu strategy workflow involves extracting proteins from biological samples, followed by enzymatic digestion and labeling using a one-tube sample preparation process. DiLeu 118d served as the boosting channel. After labeling, samples were combined for HILIC enrichment and high-pH (HpH) fractionation, followed by LC-MS/MS analysis. Reproduced or adapted with permission from ref (195). Copyright 2022, American Chemical Society.

Analyzing glycosylation in proteomics is a challenging task, primarily due to the diversity and complexity of glycan structures. Each glycosylation site may exhibit different glycan structures, thereby imparting a high degree of variability to the protein function. MS has become a key technology in glycoproteomics, enabling the identification and characterization of glycosylation sites and their attached glycan structures.^192^ However, the analysis becomes more complex due to the diversity of glycan types and the instability of the glycans. To overcome these challenges, researchers often employ enrichment strategies such as lectin affinity chromatography and HILIC to isolate glycopeptides from complex samples prior to MS analysis.^193,194^ In addition to using HILIC to enrich low-abundance glycopeptides, Wang et al. developed the boost-DiLeu strategy, which enhances the quantification of low-abundance proteins and peptides by adding an additional boosting channel, enabling more comprehensive quantitative analysis of glycopeptides (Figure 8).^195^ This method successfully quantified 1172 intact glycopeptides, including 164 glycoproteins and 18 glycopeptides, showing significant correlation with Alzheimer’s disease.

#### 4.3.3 Citrullination

Citullination, also known as deimination, is a PTM of proteins in which arginine residues in proteins are converted into citrulline. This transformation neutralizes the amino acid and increases its mass by 0.98 Da. Citrullination is catalyzed by a group of enzymes known as peptidylarginine deiminases (PADs) and has significant implications for normal physiological functions and various pathological conditions.^196^ Notably, abnormal citrullination is associated with several diseases; for instance, in rheumatoid arthritis, antibodies against citrullinated proteins are one of the disease markers and contribute to its pathogenesis.^197,198^ Citrullination is also related to other conditions such as multiple sclerosis,^199^ Alzheimer’s disease,^200^ and cancers,^201^ demonstrating its widespread impact on human health.

Identification strategies for protein citrullination include antibody- and MS-based techniques. Antibody methods identify citrulline residues and detect them through Western blotting or immunostaining procedures, but this approach struggles with low-abundance citrullinated proteins and cannot provide the specific location of citrullination sites.^202,203^ MS-based detection relies on the mass difference between the citrulline and arginine residues. However, other PTMs, such as the deamidation of asparagine or glutamine, can also lead to similar mass changes, posing difficulties in data interpretation.

To overcome these challenges, Holm et al. developed a 2,3-butanedione tagging strategy, which specifically reacts with citrulline residues to produce a 50 Da increase in monoisotopic mass.^204^ Furthermore, Li et al. combined 2,3-butanedione tagging, biotin-thiol labeling, and isobaric labeling techniques to achieve enrichment, large-scale analysis, and multiplexed quantitative analysis of protein citrullination from MCF7 cell lines in response to various types of DNA damage responses (DDR).^205^ This research highlighted significant biological processes influenced by protein citrullination and identified enhanced citrullination in RNA-binding proteins and DNA repair proteins.

### 4.4 Cross-Linking MS

Cross-linking mass spectrometry (XL-MS) is a widely used technique in the fields of proteomics and structural biology, primarily for mapping protein–protein interactions and elucidating their spatial organization. A key advantage of XL-MS is its ability to analyze large protein complexes and capture dynamic interactions of proteins in their native state, which is often challenging for other structural biology techniques like X-ray crystallography, NMR spectroscopy, and cryo-electron microscopy.^206^

XL-MS relies on specially designed cross-linking reagents that create covalent bonds between neighboring amino acid residues within a protein or between different proteins. These reagents contain two reactive groups connected by a spacer arm. When proteins contact these reagents, they form cross-links between adjacent amino acids, thereby capturing and stabilizing the spatial interaction. In XL-MS, cross-linkers are designed to react with specific side chains of amino acids. The choice of cross-linker is crucial as it determines the types of interactions and distances that can be analyzed, thereby influencing the accuracy and specificity of the protein–protein interaction map obtained.^207−209^

Quantitative cross-linking mass spectrometry (qXL-MS), combined with different labeling methods, has witnessed significant progress in studying protein–protein interactions and the dynamic changes in protein structure.^210^ This approach achieves label-free quantitation by comparing the MS signal intensities of cross-linked peptides in different samples. Specifically, it involves extracting MS1 chromatographic peak areas and employing parallel reaction monitoring (PRM) for MS2-based quantitation.^211,212^

In the SILAC quantification method, quantitation is achieved by comparing the relative abundance of light and heavy labeled cross-linked peptide pairs at the MS1 level.^213^ Chemical labeling methods, such as TMT, are used for multiplexed isotopic labeling of cross-linked peptides in multiple samples simultaneously. To overcome measurement distortion issues due to coisolation of precursor ions, accurate quantitation of cross-links is performed at the MS3 level.^214^

**Figure 9:**
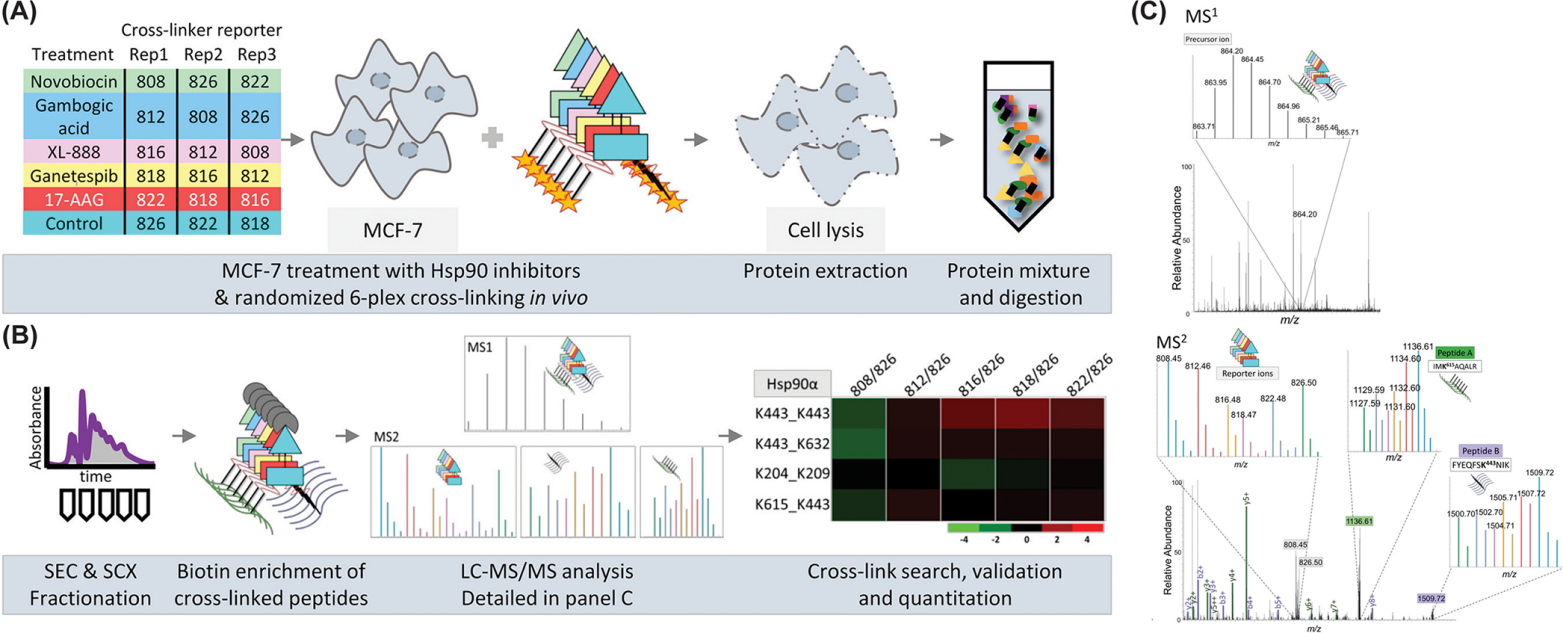
6-plex isobaric quantitative protein interaction reporter (iqPIR) experimental workflow. (A) Breast cancer cells (MCF-7) were treated with five different heat shock protein 90 (Hsp90) inhibitors, with experiments conducted in three separate replicates. The treated cells were incubated *in vivo* and subsequently cross-linked with 6-plex iqPIR. After labeling, the cells were lysed, and proteins were extracted. The labeled samples were then pooled together, followed by proteolytic digestion. (B) The combined sample was initially separated by size exclusion chromatography (SEC), followed by further fractionation using strong cation exchange (SCX). Enrichment of cross-linked peptides was then achieved through the binding of the iqPIR biotin to avidin beads. The sample underwent analysis by LC-MS. The search for and quantitation of cross-linked peptides were performed as described by Chavez et al. (C) MS1/MS2 spectra of cross-linked peptide. The MS1 spectrum shows the isotope envelope of the precursor ion, which includes all six isobaric labeled cross-linked peptides. The MS2 spectrum shows reporter ions from iqPIR and fragment ions from peptides A (b/y ions in green) and B (b/y ions in purple). Reproduced or adapted with permission from ref (216). Copyright 2022, American Chemical Society.

Recently, Chavez et al. developed the isotopic quantitative protein interaction reporter (iqPIR) technology, utilizing biotin affinity tags to enrich cross-links and incorporating stable isotopes selectively into the cross-linker for isotopic pairs of cross-linked peptides in multiple samples.^215^ This method produces unique isotopic signatures in the MS2 spectra. Currently, Wippel et al. applied the 6-plex iqPIR strategy to MCF-7 cells treated with five different Hsp90 inhibitors, 1756 cross-linking sites were successfully identified, and 1650 of these sites were quantified, revealing the interaction network dynamics of specific drug categories (Figure 9).^216^

### 4.5 Single-Cell Proteomics

Single-cell mass spectrometry (scMS) has emerged as an increasingly important tool in biological research, marking a significant shift from traditional bulk cell analysis to in-depth study at the individual cell level.^217^ Traditional proteomic methods usually rely on large numbers of cell samples, resulting in average protein expression profiles that can mask subtle differences between individual cells. These differences are crucial in areas such as cancer research,^218^ immunology,^219^ and developmental biology.^220^ Single-cell proteomics aims to reveal the complex protein composition of individual cells, providing a more refined view of cellular processes and cell-to-cell heterogeneity within cell populations.

The main challenge in single-cell proteomics is the detection and quantification of small amounts of proteins present in individual cells.^221,222^ Label-free MS is a common approach for single-cell analysis. Combined with data-independent acquisition (DIA) technology, label-free approaches can quantify up to 2000 proteins across different stages of the cell cycle.^223^ However, its major limitation is the low throughput in sample processing. Due to the time-consuming analysis process, only a limited number of cells can be processed each day, which is impractical for studies that require analysis of a large number of different cells.^224,225^ Another critical challenge in the label-free technique of single-cell proteomics is the issue of missing values. This issue primarily arises from suppression effects caused by high-abundance proteins, which complicates the accurate profiling of the entire proteome across single-cell samples. Consequently, this leads to variability in protein quantification across different samples, potentially resulting in incomplete proteome information that hampers the detection of changes in low-abundance proteins.^221^

To overcome the limitations of label-free MS, an isotopically labeled multiplexed scMS approach has been developed. The introduction of isobaric tags for iTRAQ or TMT and labeled carrier proteomes significantly enhances the MS signal, thus improving protein group coverage and ion count per peptide.^226^ By labeling proteins from carrier samples, reference samples, and individual cells with TMT, the cell ratios of these labeled samples can be precisely controlled.^227^ Schoof et al. applied a 16-plex TMTpro quantitative method, enabling the quantification of approximately 1000 proteins from multiple single-cell samples in a single LC/MS-MS analysis.^225^ This approach provides a more efficient and in-depth analytical means for single-cell level proteomic research.

### 4.6 PlexDIA

Traditional mass spectrometry often faces challenges in simultaneously achieving high throughput,^228^ in-depth proteome analysis,^229^ and minimal missing value. Employing multiplex chemical labeling methods can improve the sample throughput, reduce the MS analysis time for each sample, and reduce the number of missing values. However, common multiplex isobaric tagging methods are not compatible with DIA acquisition methods.^3^

**Figure 10:**
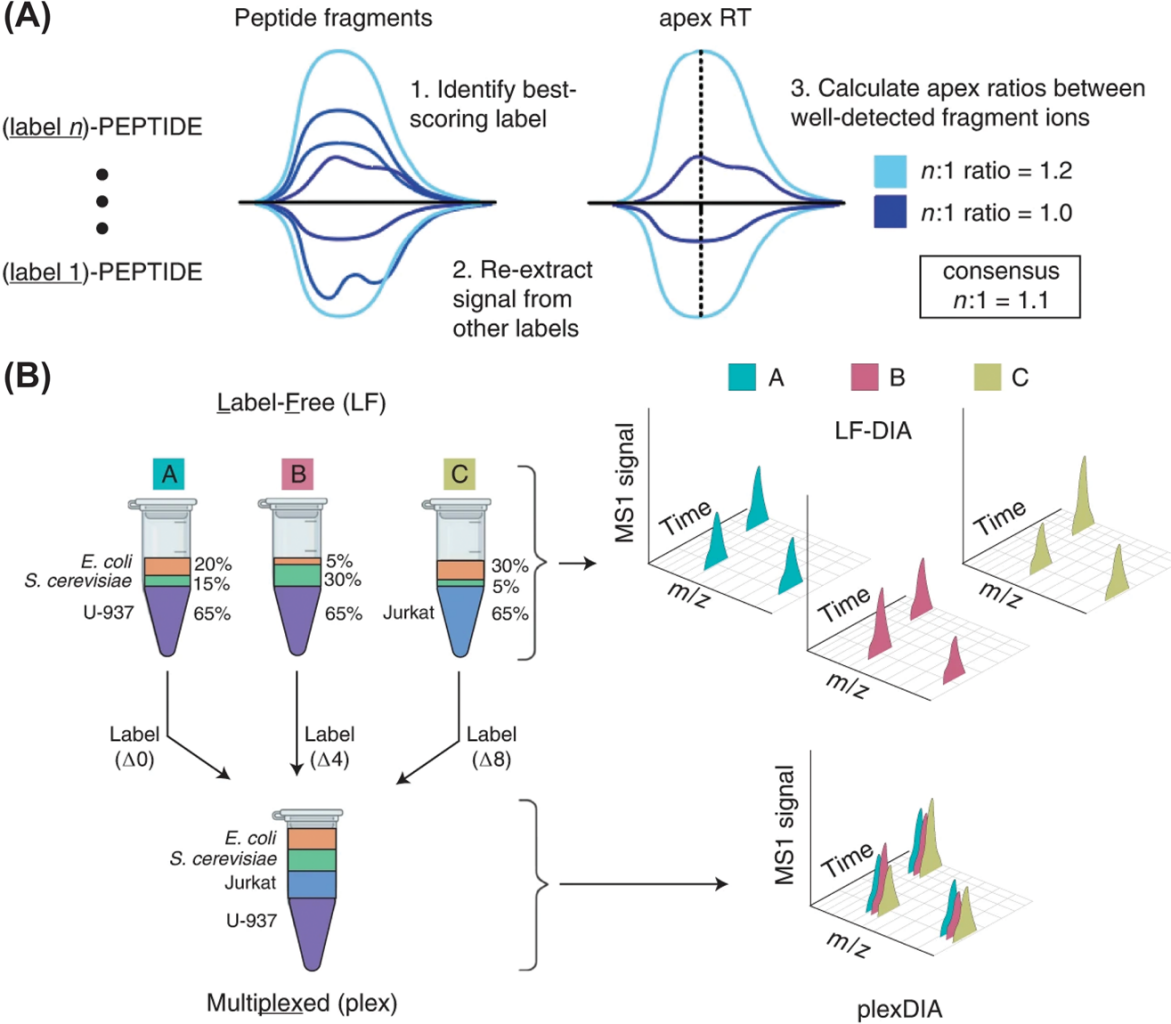
Experimental design for plexDIA analysis. (A) High confidence identified precursors from one label can be accurately transferred to other isotopologous precursors with FDR control, featuring identical retention times and known mass differences between different tags. The ratios of fragment ions were measured by calculating the most reliable isotopologous precursor against other similar precursors at their peak signal intensity, where interference is minimal. (B) Proteomes from various species and cell types were mixed in specific ratios, creating a benchmark with known protein ratios across a broad dynamic range. LF-DIA analysis utilized 500 ng from three distinct samples (A, B, and C), analyzed separately. In contrast, plexDIA combined these samples, each labeled with unique nonisobaric mass tags (mTRAQ), aiming to significantly increase quantitative data points without sacrificing accuracy. Reproduced or adapted with permission from ref (230). Copyright 2022, Springer Nature.

Derks et al. demonstrated that plexDIA, using 3-plex nonisobaric tags, enables the quantitative analysis of approximately 8000 proteins per labeled sample in 1-h gradients, significantly reducing data loss by more than 2-fold among different samples (Figure 10).^230^ This method effectively reduces the variability in protein composition between samples and across runs. In single-cell analysis, plexDIA can quantify about 1000 proteins per cell, achieving 98% data completeness in 5 min LC gradient. Therefore, plexDIA is particularly attractive for nanogram-level sample analysis, as it allows for precise and in-depth proteomic quantification analysis without the need for offline peptide separation.

### 4.7 Real-Time Analytics

Real-time analytics in proteomics is a cutting-edge approach that is significantly enhancing the capabilities of mass spectrometry-based studies.^231^ Traditional data-dependent acquisition (DDA) methods in LC-MS/MS often prioritize analyzing the most abundant or easily ionizable peptides, neglecting the less abundant ones. Additionally, DDA suffers from limitations in its dynamic range, resulting in a bias toward peptides that ionize more efficiently.

In contrast, a real-time search (RTS) in proteomics brings significant advantages by addressing these limitations. RTS rapidly matches the mass spectra against a database, identifying peptides and proteins.^232^ The real-time approach also means that peptides are not selected for MS/MS based on abundance alone. Instead, RTS can be tailored to target specific peptides of interest, including those of lower abundance, providing a more comprehensive and representative view of the proteome. This also helps to reduce sample bias and ensures a broader dynamic range of detection.

**Figure 11:**
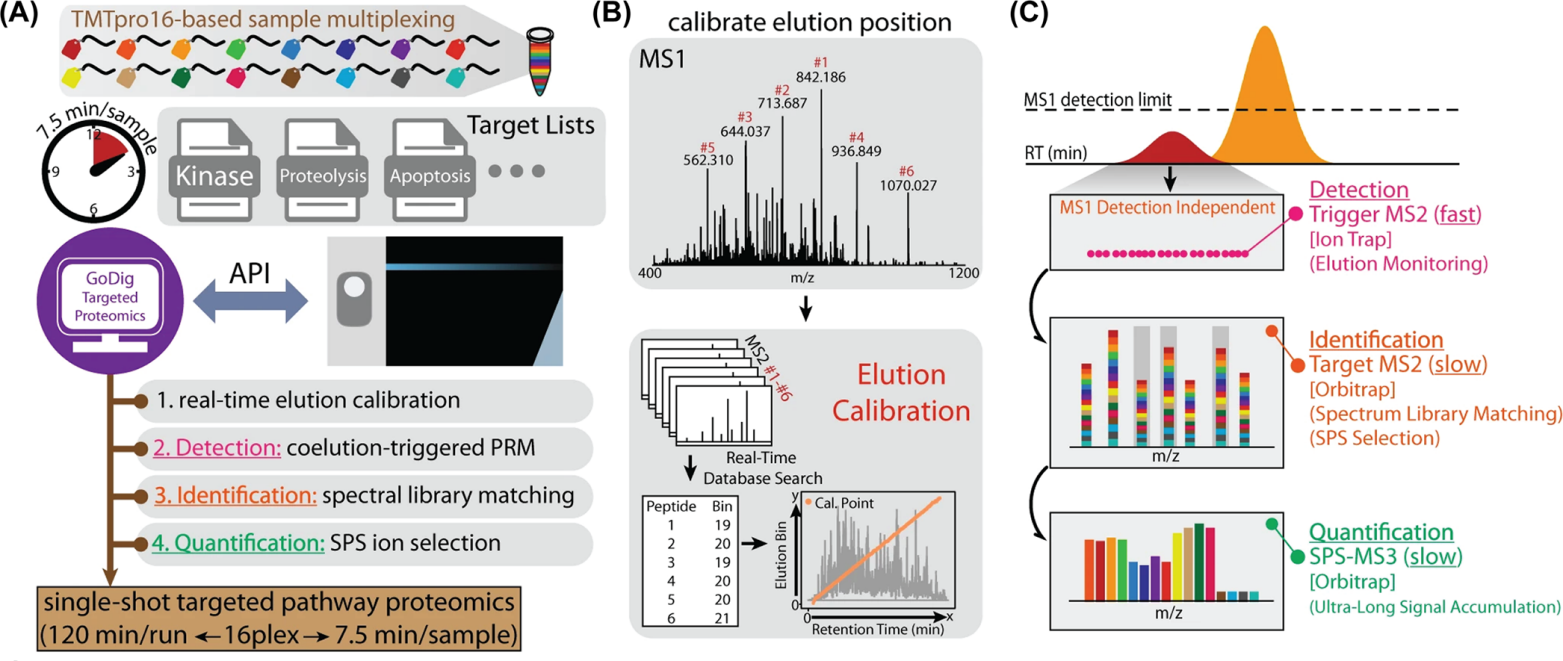
GoDig enables real-time profiling for pathway-specific measurement analysis. (A) GoDig offers multiple features that enable the multiplexed targeted proteomics, including (1) real-time elution calibration utilizing abundant MS1 peaks; (2) detection via coelution-triggered parallel reaction monitoring (PRM) scans for tracking elution; (3) identification through matching with a high-resolution spectral library; and (4) quantification using synchronous precursor selection (SPS). (B) Elution position prediction involves periodically capturing a few MS2 spectra to accurately ascertain the elution position. (C) After calibrating the elution window, GoDig initiates rapid PRM scans targeting specific peptides within the window to track their elution without MS1 detection. Upon detecting a target, GoDig captures a high-resolution MS2 scan for matching against the spectrum library and for selecting SPS ions, followed by quantification through MS3 analysis. Reprinted or adapted with permission under a Creative Commons CC-BY from ref (233). Copyright 2023, Springer Nature.

Schweppe et al. developed an advanced platform named Orbiter for real-time selection (RTS) in multiplexed synchronous precursor selection (SPS)-MS3 quantitative proteomics.^231^ This platform effectively eliminates inefficient and time-consuming MS3 scans caused by mismatches between MS2 and peptide spectra in traditional SPS-MS3 methods. Orbiter integrates RTS with error rate analysis in its online proteomics analysis pipeline, making it a rapid and efficient tool for identifying peptide spectral matches and quantifying proteins of interest. Orbiter’s RTS technology significantly enhances the speed and accuracy of proteomic analysis, capable of quantifying over 8000 proteins in half the time required by standard SPS-MS3 analysis, covering ten different proteomes. Recently, Yu et al. developed a multiplexing-based targeted pathway strategy with real-time analytics, termed GoDig.^233^ It leverages real-time analytics to pinpoint the position of target analytes presented in each sample. Without internal standard peptides, this method relies on proteomics data from past proteome-wide experiments into elution and spectral libraries. Real-time elution calibration enables the multiplexing of hundreds of target analytes into single assays. The necessary information for targeting lists derived from approximately 10,000 human proteins and 7000 mouse proteins that have been compiled as a resource. This resource is available through the GoDig assay builder Web site, facilitating the future development of pathway-specific measurement analysis (Figure 11). Overall, real-time analytics represents a significant advancement in proteomic analysis technology, improving speed and efficiency, which is particularly valuable in high-throughput analysis and large number sample processing.

## 5 Conclusion

In recent years, advancements in labeling-based proteomics have been pivotal in transforming our understanding of complex biological systems. This review thoroughly discusses the fundamental principles, applications, advantages, and limitations of various labeling strategies. This review also highlights the evolution of quantitative labeling methods, from metabolic labeling techniques such as SILAC, which integrates stable isotopes into cellular proteins, to chemical labeling approaches such as TMT and DiLeu that allow for increased throughput. Each method possesses unique advantages and inherent limitations. It is important to select the appropriate labeling strategy according to different experimental factors, including the experiment goals (discovery-based or targeted analysis), the number of samples (requirement for throughput), sample types (enabling *in vivo*/*in vitro* labeling), sample complexity (to decide if methods are needed to mitigate coisolation) and the availability of LC-MS instrument types.

Labeling methods in MS-based quantitative proteomics have become indispensable tools in bioscience applications, facilitating research in fields including cancer biomarker discovery, neuroproteomics, post-translational modifications, and the study of protein–protein interactions and single-cell proteomics. As these techniques continue to evolve, they will undoubtedly reveal new insights into the molecular mechanisms underlying health and disease, facilitating the discovery of novel biomarkers and therapeutic targets. The continued advancement and application of labeling methods in proteomics research hold great promise for enhancing our understanding of biological complexity and driving forward the frontiers of biomedical science.

## References

[ref1] Ankney J. A.; Muneer A.; Chen X. Relative and Absolute Quantitation in Mass Spectrometry–Based Proteomics. Annu. Rev. Anal. Chem. 2018, 11, 49–77. 10.1146/annurev-anchem-061516-045357. 29894226

[ref2] Pappireddi N.; Martin L.; Wühr M. A Review on Quantitative Multiplexed Proteomics. ChemBioChem. 2019, 20, 1210–1224. 10.1002/cbic.201800650. 30609196 PMC6520187

[ref3] Tian X.; Permentier H. P.; Bischoff R. Chemical isotope labeling for quantitative proteomics. Mass Spectrom. Rev. 2023, 42, 546–576. 10.1002/mas.21709. 34091937 PMC10078755

[ref4] Chen X.; et al. Quantitative Proteomics Using Isobaric Labeling: A Practical Guide. Genom., Proteom. Bioinform. 2021, 19, 689–706. 10.1016/j.gpb.2021.08.012. PMC9170757 35007772

[ref5] Lu H.; et al. DiLeu Isobaric Labeling Coupled with Limited Proteolysis Mass Spectrometry for High-Throughput Profiling of Protein Structural Changes in Alzheimer’s Disease. Anal. Chem. 2023, 95, 9746–9753. 10.1021/acs.analchem.2c05731. 37307028 PMC10330787

[ref6] Yu C.; et al. Developing a Multiplexed Quantitative Cross-Linking Mass Spectrometry Platform for Comparative Structural Analysis of Protein Complexes. Anal. Chem. 2016, 88, 10301–10308. 10.1021/acs.analchem.6b03148. 27626298 PMC5361889

[ref7] Chavez J. D.; Keller A.; Mohr J. P.; Bruce J. E. Isobaric Quantitative Protein Interaction Reporter Technology for Comparative Interactome Studies. Anal. Chem. 2020, 92, 14094–14102. 10.1021/acs.analchem.0c03128. 32969639 PMC7995634

[ref8] Wu H.; et al. Isobaric Tags for Relative and Absolute Quantitation in Proteomic Analysis of Potential Biomarkers in Invasive Cancer, Ductal Carcinoma In Situ, and Mammary Fibroadenoma. Front. Oncol. 2020, 10, 574552 10.3389/fonc.2020.574552. 33194682 PMC7640741

[ref9] Du C.; et al. Isobaric tags for relative and absolute quantitation-based proteomics reveals potential novel biomarkers for the early diagnosis of acute myocardial infarction within 3 h. Int. J. Mol. Med. 2019, 43, 1991–2004. 10.3892/ijmm.2019.4137. 30896787 PMC6443345

[ref10] Westbrook J. A.; Noirel J.; Brown J. E.; Wright P. C.; Evans C. A. Quantitation with chemical tagging reagents in biomarker studies. Proteom. Clin. Appl. 2015, 9, 295–300. 10.1002/prca.201400120. 25504339

[ref11] Dai J.; et al. TMT-labeling Proteomics of Papillary Thyroid Carcinoma Reveal Invasive Biomarkers. J. Cancer 2020, 11, 6122–6132. 10.7150/jca.47290. 32922552 PMC7477402

[ref12] Wei X.; Li L. Mass spectrometry-based proteomics and peptidomics for biomarker discovery in neurodegenerative diseases. Int. J. Clin. Exp. Pathol. 2008, 2, 132–48. 19079648 PMC2583631

[ref13] Yang K.; et al. Accelerating multiplexed profiling of protein-ligand interactions: High-throughput plate-based reactive cysteine profiling with minimal input. Cell Chem. Biol. 2024, 31, 565–576. 10.1016/j.chembiol.2023.11.015. 38118439 PMC10960705

[ref14] El-Khateeb E.; et al. Quantitative mass spectrometry-based proteomics in the era of model-informed drug development: Applications in translational pharmacology and recommendations for best practice. Pharmacol. Ther. 2019, 203, 107397 10.1016/j.pharmthera.2019.107397. 31376433

[ref15] Oda Y.; et al. Quantitative Chemical Proteomics for Identifying Candidate Drug Targets. Anal. Chem. 2003, 75, 2159–2165. 10.1021/ac026196y. 12720356

[ref16] Jiang Y.; et al. Proteomics identifies new therapeutic targets of early-stage hepatocellular carcinoma. Nature 2019, 567, 257–261. 10.1038/s41586-019-0987-8. 30814741

[ref17] Shuken S. R. An Introduction to Mass Spectrometry-Based Proteomics. J. Proteome Res. 2023, 22, 2151–2171. 10.1021/acs.jproteome.2c00838. 37260118

[ref18] Olsen J. V.; Ong S.-E.; Mann M. Trypsin Cleaves Exclusively C-terminal to Arginine and Lysine Residues*. Mol. Cell. Proteom. 2004, 3, 608–614. 10.1074/mcp.T400003-MCP200. 15034119

[ref19] Eng J. K.; McCormack A. L.; Yates J. R. An approach to correlate tandem mass spectral data of peptides with amino acid sequences in a protein database. J. Am. Soc. Mass Spectrom. 1994, 5, 976–989. 10.1016/1044-0305(94)80016-2. 24226387

[ref20] Cox J.; et al. Andromeda: A Peptide Search Engine Integrated into the MaxQuant Environment. J. Proteome Res. 2011, 10, 1794–1805. 10.1021/pr101065j. 21254760

[ref21] Muntel J.; et al. Abundance-based Classifier for the Prediction of Mass Spectrometric Peptide Detectability Upon Enrichment (PPA)* [S]. Mol. Cell. Proteom. 2015, 14, 430–440. 10.1074/mcp.M114.044321. PMC4350037 25473088

[ref22] Cox J.; Mann M. MaxQuant enables high peptide identification rates, individualized p.p.b.-range mass accuracies and proteome-wide protein quantification. Nat. Biotechnol. 2008, 26, 1367–1372. 10.1038/nbt.1511. 19029910

[ref23] Cox J.; et al. Accurate Proteome-wide Label-free Quantification by Delayed Normalization and Maximal Peptide Ratio Extraction, Termed MaxLFQ*. Mol. Cell. Proteom. 2014, 13, 2513–2526. 10.1074/mcp.M113.031591. PMC4159666 24942700

[ref24] Bondarenko P. V.; Chelius D.; Shaler T. A. Identification and Relative Quantitation of Protein Mixtures by Enzymatic Digestion Followed by Capillary Reversed-Phase Liquid Chromatography–Tandem Mass Spectrometry. Anal. Chem. 2002, 74, 4741–4749. 10.1021/ac0256991. 12349978

[ref25] Zhou Y.; Shan Y.; Zhang L.; Zhang Y. Recent advances in stable isotope labeling based techniques for proteome relative quantification. J. Chromatogr. A 2014, 1365, 1–11. 10.1016/j.chroma.2014.08.098. 25246102

[ref26] Webb-Robertson B.-J. M.; et al. Review, Evaluation, and Discussion of the Challenges of Missing Value Imputation for Mass Spectrometry-Based Label-Free Global Proteomics. J. Proteome Res. 2015, 14, 1993–2001. 10.1021/pr501138h. 25855118 PMC4776766

[ref27] Ong S.-E.; Mann M. Mass spectrometry–based proteomics turns quantitative. Nat. Chem. Biol. 2005, 1, 252–262. 10.1038/nchembio736. 16408053

[ref28] Merrill A. E.; Coon J. J. Quantifying proteomes and their post-translational modifications by stable isotope label-based mass spectrometry. Curr. Opin. Chem. Biol. 2013, 17, 779–786. 10.1016/j.cbpa.2013.06.011. 23835517 PMC3823833

[ref29] Liu J.; et al. Advances and applications of stable isotope labeling-based methods for proteome relative quantitation. TrAC Trends Anal. Chem. 2020, 124, 115815 10.1016/j.trac.2020.115815.

[ref30] Boersema P. J.; Aye T. T.; van Veen T. A. B.; Heck A. J. R.; Mohammed S. Triplex protein quantification based on stable isotope labeling by peptide dimethylation applied to cell and tissue lysates. PROTEOMICS 2008, 8, 4624–4632. 10.1002/pmic.200800297. 18850632

[ref31] Merrill A. E.; et al. NeuCode Labels for Relative Protein Quantification. Mol. Cell. Proteom. 2014, 13, 2503–2512. 10.1074/mcp.M114.040287. PMC4159665 24938287

[ref32] Oda Y.; Huang K.; Cross F. R.; Cowburn D.; Chait B. T. Accurate quantitation of protein expression and site-specific phosphorylation. Proc. Natl. Acad. Sci. U. S. A. 1999, 96, 6591–6596. 10.1073/pnas.96.12.6591. 10359756 PMC21959

[ref33] Ong S.-E.; et al. Stable Isotope Labeling by Amino Acids in Cell Culture, SILAC, as a Simple and Accurate Approach to Expression Proteomics*. Mol. Cell. Proteom. 2002, 1, 376–386. 10.1074/mcp.M200025-MCP200. 12118079

[ref34] Jiang H.; English A. M. Quantitative Analysis of the Yeast Proteome by Incorporation of Isotopically Labeled Leucine. J. Proteome Res. 2002, 1, 345–350. 10.1021/pr025523f. 12645890

[ref35] Chen X.; Wei S.; Ji Y.; Guo X.; Yang F. Quantitative proteomics using SILAC: Principles, applications, and developments. PROTEOMICS 2015, 15, 3175–3192. 10.1002/pmic.201500108. 26097186

[ref36] Zhang Y.; Fonslow B. R.; Shan B.; Baek M.-C.; Yates J. R. Protein Analysis by Shotgun/Bottom-up Proteomics. Chem. Rev. 2013, 113, 2343–2394. 10.1021/cr3003533. 23438204 PMC3751594

[ref37] Hung V.; et al. Spatially resolved proteomic mapping in living cells with the engineered peroxidase APEX2. Nat. Protoc. 2016, 11, 456–475. 10.1038/nprot.2016.018. 26866790 PMC4863649

[ref38] Dong X.; Xiong L.; Jiang X.; Wang Y. Quantitative Proteomic Analysis Reveals the Perturbation of Multiple Cellular Pathways in Jurkat-T Cells Induced by Doxorubicin. J. Proteome Res. 2010, 9, 5943–5951. 10.1021/pr1007043. 20822187 PMC2974774

[ref39] Xiong L.; Wang Y. Quantitative Proteomic Analysis Reveals the Perturbation of Multiple Cellular Pathways in HL-60 Cells Induced by Arsenite Treatment. J. Proteome Res. 2010, 9, 1129–1137. 10.1021/pr9011359. 20050688 PMC2819029

[ref40] Zhang F.; Dai X.; Wang Y. 5-Aza-2′-deoxycytidine Induced Growth Inhibition of Leukemia Cells through Modulating Endogenous Cholesterol Biosynthesis*. Mol. Cell. Proteom. 2012, 11, M111.016915-1–M111.016915-8. 10.1074/mcp.M111.016915. PMC3394957 22398368

[ref41] Meissner F.; Scheltema R. A.; Mollenkopf H.-J.; Mann M. Direct Proteomic Quantification of the Secretome of Activated Immune Cells. Science 2013, 340, 475–478. 10.1126/science.1232578. 23620052

[ref42] Liang Y.; et al. HyperSCP: Combining Isotopic and Isobaric Labeling for Higher Throughput Single-Cell Proteomics. Anal. Chem. 2023, 95, 8020–8027. 10.1021/acs.analchem.3c00906. 37167627 PMC10246935

[ref43] Geiger T.; et al. Initial Quantitative Proteomic Map of 28 Mouse Tissues Using the SILAC Mouse*. Mol. Cell. Proteom. 2013, 12, 1709–1722. 10.1074/mcp.M112.024919. PMC3675825 23436904

[ref44] Zanivan S.; Krueger M.; Mann M. Integrin and Cell Adhesion Molecules, Methods and Protocols. Methods Mol. Biol. 2011, 757, 435–450. 10.1007/978-1-61779-166-6_25. 21909926

[ref45] Krüger M.; et al. SILAC Mouse for Quantitative Proteomics Uncovers Kindlin-3 as an Essential Factor for Red Blood Cell Function. Cell 2008, 134, 353–364. 10.1016/j.cell.2008.05.033. 18662549

[ref46] Sury M. D.; Chen J.-X.; Selbach M. The SILAC Fly Allows for Accurate Protein Quantification in Vivo *. Mol. Cell. Proteom. 2010, 9, 2173–2183. 10.1074/mcp.M110.000323. PMC2953914 20525996

[ref47] Miyagi M.; Rao K. C. S. Proteolytic 18O-labeling strategies for quantitative proteomics. Mass Spectrom. Rev. 2007, 26, 121–136. 10.1002/mas.20116. 17086517

[ref48] Zhao Y.; et al. Combination of Improved 18O Incorporation and Multiple Reaction Monitoring: A Universal Strategy for Absolute Quantitative Verification of Serum Candidate Biomarkers of Liver Cancer. J. Proteome Res. 2010, 9, 3319–3327. 10.1021/pr9011969. 20420461

[ref49] Zhang S.; et al. Integrated platform with a combination of online digestion and 18 O labeling for proteome quantification via an immobilized trypsin microreactor. Analyst 2015, 140, 5227–5234. 10.1039/C5AN00887E. 26063120

[ref50] Gygi S. P.; et al. Quantitative analysis of complex protein mixtures using isotope-coded affinity tags. Nat. Biotechnol. 1999, 17, 994–999. 10.1038/13690. 10504701

[ref51] Han D. K.; Eng J.; Zhou H.; Aebersold R. Quantitative profiling of differentiation-induced microsomal proteins using isotope-coded affinity tags and mass spectrometry. Nat. Biotechnol. 2001, 19, 946–951. 10.1038/nbt1001-946. 11581660 PMC1444949

[ref52] Hsu J.-L.; Huang S.-Y.; Chow N.-H.; Chen S.-H. Stable-Isotope Dimethyl Labeling for Quantitative Proteomics. Anal. Chem. 2003, 75, 6843–6852. 10.1021/ac0348625. 14670044

[ref53] Boersema P. J.; Raijmakers R.; Lemeer S.; Mohammed S.; Heck A. J. R. Multiplex peptide stable isotope dimethyl labeling for quantitative proteomics. Nat. Protoc. 2009, 4, 484–494. 10.1038/nprot.2009.21. 19300442

[ref54] Fu Q.; Li L. De Novo Sequencing of Neuropeptides Using Reductive Isotopic Methylation and Investigation of ESI QTOF MS/MS Fragmentation Pattern of Neuropeptides with N-Terminal Dimethylation. Anal. Chem. 2005, 77, 7783–7795. 10.1021/ac051324e. 16316189

[ref55] Wu Y.; et al. Five-plex isotope dimethyl labeling for quantitative proteomics. Chem. Commun. 2014, 50, 1708–1710. 10.1039/c3cc47998f. 24394284

[ref56] Paulo J. A.; Gygi S. P. mTMT: An Alternative, Nonisobaric, Tandem Mass Tag Allowing for Precursor-Based Quantification. Anal. Chem. 2019, 91, 12167–12172. 10.1021/acs.analchem.9b03162. 31490667 PMC7294932

[ref57] Kang U.-B.; Yeom J.; Kim H.; Lee C. Quantitative Analysis of mTRAQ-Labeled Proteome Using Full MS Scans. J. Proteome Res. 2010, 9, 3750–3758. 10.1021/pr9011014. 20465265

[ref58] Greer T.; Lietz C. B.; Xiang F.; Li L. Novel isotopic N,N-Dimethyl Leucine (iDiLeu) Reagents Enable Absolute Quantification of Peptides and Proteins Using a Standard Curve Approach. J. Am. Soc. Mass Spectrom. 2015, 26, 107–119. 10.1007/s13361-014-1012-y. 25377360 PMC4276538

[ref59] Hebert A. S.; et al. Neutron-encoded mass signatures for multiplexed proteome quantification. Nat. Methods 2013, 10, 332–334. 10.1038/nmeth.2378. 23435260 PMC3612390

[ref60] Hebert A. S.; et al. Amine-reactive Neutron-encoded Labels for Highly Plexed Proteomic Quantitation*. Mol. Cell. Proteom. 2013, 12, 3360–3369. 10.1074/mcp.M113.032011. PMC3820946 23882030

[ref61] Frost D. C.; Buchberger A. R.; Li L. Mass Defect-Based Dimethyl Pyrimidinyl Ornithine (DiPyrO) Tags for Multiplex Quantitative Proteomics. Anal. Chem. 2017, 89, 10798–10805. 10.1021/acs.analchem.7b02098. 28795795 PMC7491675

[ref62] Hao L.; et al. Mass Defect-Based N,N-Dimethyl Leucine Labels for Quantitative Proteomics and Amine Metabolomics of Pancreatic Cancer Cells. Anal. Chem. 2017, 89, 1138–1146. 10.1021/acs.analchem.6b03482. 28194987 PMC5338727

[ref63] Zhong X.; Frost D. C.; Li L. High-Resolution Enabled 5-plex Mass Defect-Based N,N-Dimethyl Leucine Tags for Quantitative Proteomics. Anal. Chem. 2019, 91, 7991–7995. 10.1021/acs.analchem.9b01691. 31135137 PMC7170397

[ref64] Ma M.; et al. 6-Plex mdSUGAR Isobaric-Labeling Guide Fingerprint Embedding for Glycomics Analysis. Anal. Chem. 2023, 95, 17637–17645. 10.1021/acs.analchem.3c03342. 37982459 PMC10794169

[ref65] Di Y.; et al. MdCDPM: A Mass Defect-Based Chemical-Directed Proteomics Method for Targeted Analysis of Intact Sialylglycopeptides. Anal. Chem. 2019, 91, 9986–9992. 10.1021/acs.analchem.9b01798. 31313914

[ref66] Wang Q.; et al. mNeuCode Empowers Targeted Proteome Analysis of Arginine Dimethylation. Anal. Chem. 2023, 95, 3684–3693. 10.1021/acs.analchem.2c04648. 36757215

[ref67] Arul A. B.; Robinson R. A. S. Sample Multiplexing Strategies in Quantitative Proteomics. Anal. Chem. 2019, 91, 178–189. 10.1021/acs.analchem.8b05626. 30525468 PMC6951796

[ref68] McAlister G. C.; et al. Increasing the Multiplexing Capacity of TMTs Using Reporter Ion Isotopologues with Isobaric Masses. Anal. Chem. 2012, 84, 7469–7478. 10.1021/ac301572t. 22880955 PMC3715028

[ref69] Frost D. C.; Greer T.; Li L. High-Resolution Enabled 12-Plex DiLeu Isobaric Tags for Quantitative Proteomics. Anal. Chem. 2015, 87, 1646–1654. 10.1021/ac503276z. 25405479 PMC4318621

[ref70] Thompson A.; et al. Tandem Mass Tags: A Novel Quantification Strategy for Comparative Analysis of Complex Protein Mixtures by MS/MS. Anal. Chem. 2003, 75, 1895–1904. 10.1021/ac0262560. 12713048

[ref71] Ross P. L.; et al. Multiplexed Protein Quantitation in Saccharomyces cerevisiae Using Amine-reactive Isobaric Tagging Reagents*. Mol. Cell. Proteom. 2004, 3, 1154–1169. 10.1074/mcp.M400129-MCP200. 15385600

[ref72] Mertins P.; et al. iTRAQ Labeling is Superior to mTRAQ for Quantitative Global Proteomics and Phosphoproteomics*. Mol. Cell. Proteom. 2012, 11, M111.014423 10.1074/mcp.M111.014423. PMC3433912 22210691

[ref73] Choe L.; et al. 8-Plex quantitation of changes in cerebrospinal fluid protein expression in subjects undergoing intravenous immunoglobulin treatment for Alzheimer’s disease. PROTEOMICS 2007, 7, 3651–3660. 10.1002/pmic.200700316. 17880003 PMC3594777

[ref74] Dayon L.; et al. Relative Quantification of Proteins in Human Cerebrospinal Fluids by MS/MS Using 6-Plex Isobaric Tags. Anal. Chem. 2008, 80, 2921–2931. 10.1021/ac702422x. 18312001

[ref75] Li J.; et al. TMTpro reagents: a set of isobaric labeling mass tags enables simultaneous proteome-wide measurements across 16 samples. Nat. Methods 2020, 17, 399–404. 10.1038/s41592-020-0781-4. 32203386 PMC7302421

[ref76] Li J.; et al. TMTpro-18plex: The Expanded and Complete Set of TMTpro Reagents for Sample Multiplexing. J. Proteome Res. 2021, 20, 2964–2972. 10.1021/acs.jproteome.1c00168. 33900084 PMC8210943

[ref77] Thompson A.; et al. TMTpro: Design, Synthesis, and Initial Evaluation of a Proline-Based Isobaric 16-Plex Tandem Mass Tag Reagent Set. Anal. Chem. 2019, 91, 15941–15950. 10.1021/acs.analchem.9b04474. 31738517

[ref78] Hsu J.-L.; Huang S.-Y.; Shiea J.-T.; Huang W.-Y.; Chen S.-H. Beyond Quantitative Proteomics: Signal Enhancement of the a1 Ion as a Mass Tag for Peptide Sequencing Using Dimethyl Labeling. J. Proteome Res. 2005, 4, 101–108. 10.1021/pr049837+. 15707364

[ref79] Colzani M.; Schütz F.; Potts A.; Waridel P.; Quadroni M. Relative Protein Quantification by Isobaric SILAC with Immonium Ion Splitting (ISIS)*. Mol. Cell. Proteom. 2008, 7, 927–937. 10.1074/mcp.M700440-MCP200. 18165257

[ref80] Xiang F.; Ye H.; Chen R.; Fu Q.; Li L. N,N-Dimethyl Leucines as Novel Isobaric Tandem Mass Tags for Quantitative Proteomics and Peptidomics. Anal. Chem. 2010, 82, 2817–2825. 10.1021/ac902778d. 20218596 PMC2859709

[ref81] Frost D. C.; Feng Y.; Li L. 21-plex DiLeu Isobaric Tags for High-Throughput Quantitative Proteomics. Anal. Chem. 2020, 92, 8228–8234. 10.1021/acs.analchem.0c00473. 32401496 PMC7306224

[ref82] Frost D. C.; Li L. Quantitative Proteomics by Mass Spectrometry. Methods Mol. Biol. 2016, 1410, 169–194. 10.1007/978-1-4939-3524-6_10. 26867744

[ref83] Sivanich M. K.; Gu T.; Tabang D. N.; Li L. Recent advances in isobaric labeling and applications in quantitative proteomics. PROTEOMICS 2022, 22, 2100256 10.1002/pmic.202100256. 35687565 PMC9787039

[ref84] Frost D. C.; Greer T.; Xiang F.; Liang Z.; Li L. Development and characterization of novel 8-plex DiLeu isobaric labels for quantitative proteomics and peptidomics. Rapid Commun. Mass Spectrom. 2015, 29, 1115–1124. 10.1002/rcm.7201. 25981542 PMC4837894

[ref85] Ren Y.; et al. Reagents for Isobaric Labeling Peptides in Quantitative Proteomics. Anal. Chem. 2018, 90, 12366–12371. 10.1021/acs.analchem.8b00321. 30260629

[ref86] Ning X.; et al. New Set of Isobaric Labeling Reagents for Quantitative 16Plex Proteomics. Anal. Chem. 2023, 95, 5788–5795. 10.1021/acs.analchem.3c00235. 36958307

[ref87] Dayon L.; Affolter M. Progress and pitfalls of using isobaric mass tags for proteome profiling. Expert Rev. Proteom. 2020, 17, 149–161. 10.1080/14789450.2020.1731309. 32067523

[ref88] Frost D. C.; Rust C. J.; Robinson R. A. S.; Li L. Increased N,N-Dimethyl Leucine Isobaric Tag Multiplexing by a Combined Precursor Isotopic Labeling and Isobaric Tagging Approach. Anal. Chem. 2018, 90, 10664–10669. 10.1021/acs.analchem.8b01301. 30095893 PMC6314838

[ref89] Evans A. R.; Robinson R. A. S. Global combined precursor isotopic labeling and isobaric tagging (cPILOT) approach with selective MS3 acquisition. PROTEOMICS 2013, 13, 3267–3272. 10.1002/pmic.201300198. 24124127

[ref90] King C. D.; Dudenhoeffer J. D.; Gu L.; Evans A. R.; Robinson R. A. S. Enhanced Sample Multiplexing of Tissues Using Combined Precursor Isotopic Labeling and Isobaric Tagging (cPILOT). J. Vis. Exp. 2017, 123, e55406 10.3791/55406-v. PMC5565145 28518113

[ref91] Xing S.; Pai A.; Wu R.; Lu Y. NHS-Ester Tandem Labeling in One Pot Enables 48-Plex Quantitative Proteomics. Anal. Chem. 2021, 93, 12827–12832. 10.1021/acs.analchem.1c01314. 34529408

[ref92] Wu Z.; Shen Y.; Zhang X. TAG-TMTpro, a Hyperplexing Quantitative Approach for High-Throughput Proteomic Studies. Anal. Chem. 2022, 94, 12565–12569. 10.1021/acs.analchem.2c02099. 36066113

[ref93] Wu Z.; Huang X.; Huang L.; Zhang X. 102-Plex Approach for Accurate and Multiplexed Proteome Quantification. Anal. Chem. 2024, 96, 1402–1409. 10.1021/acs.analchem.3c03036. 38215345

[ref94] Wu Z.; Xiang W.; Huang L.; Li S.; Zhang X. Hyperplexing Approaches for up to 45-Plex Quantitative Proteomic Analysis. Anal. Chem. 2023, 95, 5169–5175. 10.1021/acs.analchem.3c00237. 36917635

[ref95] Sun H.; et al. 29-Plex tandem mass tag mass spectrometry enabling accurate quantification by interference correction. PROTEOMICS 2022, 22, e2100243 10.1002/pmic.202100243. 35723178 PMC9588555

[ref96] Wang Z.; et al. 27-Plex Tandem Mass Tag Mass Spectrometry for Profiling Brain Proteome in Alzheimer’s Disease. Anal. Chem. 2020, 92, 7162–7170. 10.1021/acs.analchem.0c00655. 32343560 PMC8176402

[ref97] Bowser B. L.; Robinson R. A. S. Enhanced Multiplexing Technology for Proteomics. Annu. Rev. Anal. Chem. 2023, 16, 379–400. 10.1146/annurev-anchem-091622-092353. 36854207

[ref98] Yu Q.; et al. Benchmarking the Orbitrap Tribrid Eclipse for Next Generation Multiplexed Proteomics. Anal. Chem. 2020, 92, 6478–6485. 10.1021/acs.analchem.9b05685. 32250601 PMC7295122

[ref99] McAlister G. C.; et al. MultiNotch MS3 Enables Accurate, Sensitive, and Multiplexed Detection of Differential Expression across Cancer Cell Line Proteomes. Anal. Chem. 2014, 86, 7150–7158. 10.1021/ac502040v. 24927332 PMC4215866

[ref100] Wenger C. D.; et al. Gas-phase purification enables accurate, multiplexed proteome quantification with isobaric tagging. Nat. Methods 2011, 8, 933–935. 10.1038/nmeth.1716. 21963608 PMC3205195

[ref101] Paulo J. A.; O’Connell J. D.; Gygi S. P. A Triple Knockout (TKO) Proteomics Standard for Diagnosing Ion Interference in Isobaric Labeling Experiments. J. Am. Soc. Mass Spectrom. 2016, 27, 1620–1625. 10.1007/s13361-016-1434-9. 27400695 PMC5018445

[ref102] Gygi J. P.; et al. A Triple Knockout Isobaric-Labeling Quality Control Platform with an Integrated Online Database Search. J. Am. Soc. Mass Spectrom. 2020, 31, 1344–1349. 10.1021/jasms.0c00029. 32202424 PMC7332369

[ref103] Savitski M. M.; et al. Delayed Fragmentation and Optimized Isolation Width Settings for Improvement of Protein Identification and Accuracy of Isobaric Mass Tag Quantification on Orbitrap-Type Mass Spectrometers. Anal. Chem. 2011, 83, 8959–8967. 10.1021/ac201760x. 22017476

[ref104] Next-generation TMTpro reagents for increased sample multiplexing. https://assets.thermofisher.com/TFS-Assets/BID/Application-Notes/next-generation-tmtpro-reagents-multiplexing-app-note.pdf (March 29, 2024).

[ref105] TMT/TMTpro Instrument Acquisition.https://assets.thermofisher.com/TFS-Assets/BID/Reference-Materials/tmt-tmtpro-instrument-acquisition.pdf (March 29, 2024).

[ref106] Yang Y.; et al. Evaluation of Different Multidimensional LC–MS/MS Pipelines for Isobaric Tags for Relative and Absolute Quantitation (iTRAQ)-Based Proteomic Analysis of Potato Tubers in Response to Cold Storage. J. Proteome Res. 2011, 10, 4647–4660. 10.1021/pr200455s. 21842911

[ref107] Kelstrup C. D.; et al. Rapid and Deep Proteomes by Faster Sequencing on a Benchtop Quadrupole Ultra-High-Field Orbitrap Mass Spectrometer. J. Proteome Res. 2014, 13, 6187–6195. 10.1021/pr500985w. 25349961

[ref108] Niu M.; et al. Extensive Peptide Fractionation and y 1 Ion-Based Interference Detection Method for Enabling Accurate Quantification by Isobaric Labeling and Mass Spectrometry. Anal. Chem. 2017, 89, 2956–2963. 10.1021/acs.analchem.6b04415. 28194965 PMC5467445

[ref109] Peng J.; Elias J. E.; Thoreen C. C.; Licklider L. J.; Gygi S. P. Evaluation of Multidimensional Chromatography Coupled with Tandem Mass Spectrometry (LC/LC–MS/MS) for Large-Scale Protein Analysis: The Yeast Proteome. J. Proteome Res. 2003, 2, 43–50. 10.1021/pr025556v. 12643542

[ref110] Ow S. Y.; Salim M.; Noirel J.; Evans C.; Wright P. C. & Wright, Phillip. C. Minimising iTRAQ ratio compression through understanding LC-MS elution dependence and high-resolution HILIC fractionation. PROTEOMICS 2011, 11, 2341–2346. 10.1002/pmic.201000752. 21548092

[ref111] Mant C. T.; et al. Peptide Characterization and Application Protocols. Pept. Charact. Appl. Protoc. 2007, 386, 3–55. 10.1007/978-1-59745-430-8_1.

[ref112] Ting L.; Rad R.; Gygi S. P.; Haas W. MS3 eliminates ratio distortion in isobaric multiplexed quantitative proteomics. Nat. Methods 2011, 8, 937–940. 10.1038/nmeth.1714. 21963607 PMC3205343

[ref113] Li M.; Ma M.; Li L. Development of novel isobaric tags enables accurate and sensitive multiplexed proteomics using complementary ions. Anal. Bioanal. Chem. 2023, 415, 6951–6960. 10.1007/s00216-023-04877-3. 37530794 PMC10729713

[ref114] Erickson B. K.; et al. Active Instrument Engagement Combined with a Real-Time Database Search for Improved Performance of Sample Multiplexing Workflows. J. Proteome Res. 2019, 18, 1299–1306. 10.1021/acs.jproteome.8b00899. 30658528 PMC7081948

[ref115] Schweppe D. K.; et al. Full-Featured, Real-Time Database Searching Platform Enables Fast and Accurate Multiplexed Quantitative Proteomics. J. Proteome Res. 2020, 19, 2026–2034. 10.1021/acs.jproteome.9b00860. 32126768 PMC7295121

[ref116] Shliaha P. V.; et al. Additional Precursor Purification in Isobaric Mass Tagging Experiments by Traveling Wave Ion Mobility Separation (TWIMS). J. Proteome Res. 2014, 13, 3360–3369. 10.1021/pr500220g. 24854137

[ref117] Ogata K.; Ishihama Y. Extending the Separation Space with Trapped Ion Mobility Spectrometry Improves the Accuracy of Isobaric Tag-Based Quantitation in Proteomic LC/MS/MS. Anal. Chem. 2020, 92, 8037–8040. 10.1021/acs.analchem.0c01695. 32441512

[ref118] Delafield D. G.; Lu G.; Kaminsky C. J.; Li L. High-end ion mobility mass spectrometry: A current review of analytical capacity in omics applications and structural investigations. TrAC Trends Anal. Chem. 2022, 157, 116761 10.1016/j.trac.2022.116761.

[ref119] Schweppe D. K.; et al. Characterization and Optimization of Multiplexed Quantitative Analyses Using High-Field Asymmetric-Waveform Ion Mobility Mass Spectrometry. Anal. Chem. 2019, 91, 4010–4016. 10.1021/acs.analchem.8b05399. 30672687 PMC6993951

[ref120] Pfammatter S.; Bonneil E.; Thibault P. Improvement of Quantitative Measurements in Multiplex Proteomics Using High-Field Asymmetric Waveform Spectrometry. J. Proteome Res. 2016, 15, 4653–4665. 10.1021/acs.jproteome.6b00745. 27723353

[ref121] Pfammatter S.; et al. A Novel Differential Ion Mobility Device Expands the Depth of Proteome Coverage and the Sensitivity of Multiplex Proteomic Measurements*. Mol. Cell. Proteom. 2018, 17, 2051–2067. 10.1074/mcp.TIR118.000862. PMC6166672 30007914

[ref122] Fang P.; et al. Evaluation and Optimization of High-Field Asymmetric Waveform Ion-Mobility Spectrometry for Multiplexed Quantitative Site-Specific N-Glycoproteomics. Anal. Chem. 2021, 93, 8846–8855. 10.1021/acs.analchem.1c00802. 34133129

[ref123] Rangel-Angarita V.; et al. False-Positive Glycopeptide Identification via In-FAIMS Fragmentation. JACS Au 2023, 3, 2498–2509. 10.1021/jacsau.3c00264. 37772174 PMC10523363

[ref124] Koehler C. J.; Strozynski M.; Kozielski F.; Treumann A.; Thiede B. Isobaric Peptide Termini Labeling for MS/MS-Based Quantitative Proteomics. J. Proteome Res. 2009, 8, 4333–4341. 10.1021/pr900425n. 19655813

[ref125] Koehler C. J.; Arntzen M. Ø.; de Souza G. A.; Thiede B. An Approach for Triplex-Isobaric Peptide Termini Labeling (Triplex-IPTL). Anal. Chem. 2013, 85, 2478–2485. 10.1021/ac3035508. 23316706

[ref126] Koehler C. J.; Arntzen M. Ø.; Strozynski M.; Treumann A.; Thiede B. Isobaric Peptide Termini Labeling Utilizing Site-Specific N-Terminal Succinylation. Anal. Chem. 2011, 83, 4775–4781. 10.1021/ac200229w. 21528900

[ref127] Liu J.; et al. A Multiplex Fragment-Ion-Based Method for Accurate Proteome Quantification. Anal. Chem. 2019, 91, 3921–3928. 10.1021/acs.analchem.8b04806. 30789256

[ref128] Arntzen M. Ø.; et al. IsobariQ: Software for Isobaric Quantitative Proteomics using IPTL, iTRAQ, and TMT. J. Proteome Res. 2011, 10, 913–920. 10.1021/pr1009977. 21067241

[ref129] Xie L.; et al. ITMSQ: A software tool for N- and C-terminal fragment ion pairs based isobaric tandem mass spectrometry quantification. PROTEOMICS 2015, 15, 3755–3764. 10.1002/pmic.201400513. 26349451

[ref130] Zhou Y.; et al. Mass Defect-Based Pseudo-Isobaric Dimethyl Labeling for Proteome Quantification. Anal. Chem. 2013, 85, 10658–10663. 10.1021/ac402834w. 24180428

[ref131] Tian X.; de Vries M. P.; Permentier H. P.; Bischoff R. A Collision-Induced Dissociation Cleavable Isobaric Tag for Peptide Fragment Ion-Based Quantification in Proteomics. J. Proteome Res. 2020, 19, 3817–3824. 10.1021/acs.jproteome.0c00371. 32786690 PMC7476077

[ref132] Liu J.; et al. A1 Ions: Peptide-Specific and Intensity-Enhanced Fragment Ions for Accurate and Multiplexed Proteome Quantitation. Anal. Chem. 2022, 94, 7637–7646. 10.1021/acs.analchem.2c00876. 35590477

[ref133] Wang Z.; et al. Segmented MS/MS acquisition of a1 ion-based strategy for in-depth proteome quantitation. Anal. Chim. Acta 2022, 1232, 340491 10.1016/j.aca.2022.340491. 36257755

[ref134] Tian X.; de Vries M. P.; Permentier H. P.; Bischoff R. A Versatile Isobaric Tag Enables Proteome Quantification in Data-Dependent and Data-Independent Acquisition Modes. Anal. Chem. 2020, 92, 16149–16157. 10.1021/acs.analchem.0c03858. 33256395 PMC7745205

[ref135] Wühr M.; et al. Accurate Multiplexed Proteomics at the MS2 Level Using the Complement Reporter Ion Cluster. Anal. Chem. 2012, 84, 9214–9221. 10.1021/ac301962s. 23098179 PMC3771681

[ref136] Sonnett M.; Yeung E.; Wühr M. Accurate, Sensitive, and Precise Multiplexed Proteomics Using the Complement Reporter Ion Cluster. Anal. Chem. 2018, 90, 5032–5039. 10.1021/acs.analchem.7b04713. 29522331 PMC6220677

[ref137] Johnson A.; Stadlmeier M.; Wühr M. TMTpro Complementary Ion Quantification Increases Plexing and Sensitivity for Accurate Multiplexed Proteomics at the MS2 Level. J. Proteome Res. 2021, 20, 3043–3052. 10.1021/acs.jproteome.0c00813. 33929851 PMC8330405

[ref138] Virreira Winter S. V.; et al. EASI-tag enables accurate multiplexed and interference-free MS2-based proteome quantification. Nat. Methods 2018, 15, 527–530. 10.1038/s41592-018-0037-8. 29915187

[ref139] Kozhinov A. N.; et al. Super-Resolution Mass Spectrometry Enables Rapid, Accurate, and Highly Multiplexed Proteomics at the MS2 Level. Anal. Chem. 2023, 95, 3712–3719. 10.1021/acs.analchem.2c04742. 36749928 PMC9974827

[ref140] Zaman M.; et al. Dissecting Detergent-Insoluble Proteome in Alzheimer’s Disease by TMTc-Corrected Quantitative Mass Spectrometry. Mol. Cell. Proteom. 2023, 22, 100608 10.1016/j.mcpro.2023.100608. PMC10392608 37356496

[ref141] Stadlmeier M.; Bogena J.; Wallner M.; Wühr M.; Carell T. A Sulfoxide-Based Isobaric Labelling Reagent for Accurate Quantitative Mass Spectrometry. Angew. Chem., Int. Ed. 2018, 57, 2958–2962. 10.1002/anie.201708867. 29316131

[ref142] Sager R. Expression genetics in cancer: Shifting the focus from DNA to RNA. Proc. Natl. Acad. Sci. U. S. A. 1997, 94, 952–955. 10.1073/pnas.94.3.952. 9023363 PMC19620

[ref143] Gygi S. P.; Corthals G. L.; Zhang Y.; Rochon Y.; Aebersold R. Evaluation of two-dimensional gel electrophoresis-based proteome analysis technology. Proc. Natl. Acad. Sci. U. S. A. 2000, 97, 9390–9395. 10.1073/pnas.160270797. 10920198 PMC16874

[ref144] Lee P. Y.; Saraygord-Afshari N.; Low T. Y. The evolution of two-dimensional gel electrophoresis - from proteomics to emerging alternative applications. J. Chromatogr. A 2020, 1615, 460763 10.1016/j.chroma.2019.460763. 31836310

[ref145] Sarhadi V. K.; Armengol G. Molecular Biomarkers in Cancer. Biomolecules 2022, 12, 1021 10.3390/biom12081021. 35892331 PMC9331210

[ref146] Kwon Y. W.; et al. Application of Proteomics in Cancer: Recent Trends and Approaches for Biomarkers Discovery. Front. Med. 2021, 8, 747333 10.3389/fmed.2021.747333. PMC8492935 34631760

[ref147] Dutta H.; Jain N. Post-translational modifications and their implications in cancer. Front. Oncol. 2023, 13, 1240115 10.3389/fonc.2023.1240115. 37795435 PMC10546021

[ref148] Gao Q.; et al. Integrated Proteogenomic Characterization of HBV-Related Hepatocellular Carcinoma. Cell 2019, 179, 561–577. 10.1016/j.cell.2019.08.052. 31585088

[ref149] Clark D. J.; et al. Impact of Increased FUT8 Expression on the Extracellular Vesicle Proteome in Prostate Cancer Cells. J. Proteome Res. 2020, 19, 2195–2205. 10.1021/acs.jproteome.9b00578. 32378902 PMC7345075

[ref150] Yan B.; et al. iTRAQ-based Comparative Serum Proteomic Analysis of Prostate Cancer Patients with or without Bone Metastasis. J. Cancer 2019, 10, 4165–4177. 10.7150/jca.33497. 31413735 PMC6691707

[ref151] Lin C.; et al. ITRAQ-based quantitative proteomics reveals apolipoprotein A-I and transferrin as potential serum markers in CA19–9 negative pancreatic ductal adenocarcinoma. Medicine 2016, 95, e4527 10.1097/MD.0000000000004527. 27495108 PMC4979862

[ref152] Perera R. M.; et al. Transcriptional control of autophagy–lysosome function drives pancreatic cancer metabolism. Nature 2015, 524, 361–365. 10.1038/nature14587. 26168401 PMC5086585

[ref153] Asleh K.; et al. Proteomic analysis of archival breast cancer clinical specimens identifies biological subtypes with distinct survival outcomes. Nat. Commun. 2022, 13, 896 10.1038/s41467-022-28524-0. 35173148 PMC8850446

[ref154] Liu F.; et al. PKM2 methylation by CARM1 activates aerobic glycolysis to promote tumorigenesis. Nat. Cell Biol. 2017, 19, 1358–1370. 10.1038/ncb3630. 29058718 PMC5683091

[ref155] Musrap N.; et al. Comparative Proteomics of Ovarian Cancer Aggregate Formation Reveals an Increased Expression of Calcium-activated Chloride Channel Regulator 1 (CLCA1)*. J. Biol. Chem. 2015, 290, 17218–17227. 10.1074/jbc.M115.639773. 26004777 PMC4498061

[ref156] Zhang W.; Ou X.; Wu X. Proteomics profiling of plasma exosomes in epithelial ovarian cancer: A potential role in the coagulation cascade, diagnosis and prognosis. Int. J. Oncol. 2019, 54, 1719–1733. 10.3892/ijo.2019.4742. 30864689 PMC6438431

[ref157] Chen X.; et al. A novel USP9X substrate TTK contributes to tumorigenesis in non-small-cell lung cancer. Theranostics 2018, 8, 2348–2360. 10.7150/thno.22901. 29721084 PMC5928894

[ref158] Wang C.-I.; et al. Quantitative Proteomics Reveals a Novel Role of Karyopherin Alpha 2 in Cell Migration through the Regulation of Vimentin–pErk Protein Complex Levels in Lung Cancer. J. Proteome Res. 2015, 14, 1739–1751. 10.1021/pr501097a. 25728791

[ref159] Bayés A.; Grant S. G. N. Neuroproteomics: understanding the molecular organization and complexity of the brain. Nat. Rev. Neurosci. 2009, 10, 635–646. 10.1038/nrn2701. 19693028

[ref160] Jain M.; et al. Unveiling the Molecular Footprint: Proteome-Based Biomarkers for Alzheimer’s Disease. Proteomes 2023, 11, 33 10.3390/proteomes11040033. 37873875 PMC10594437

[ref161] Johnson E. C. B.; et al. Large-scale deep multi-layer analysis of Alzheimer’s disease brain reveals strong proteomic disease-related changes not observed at the RNA level. Nat. Neurosci. 2022, 25, 213–225. 10.1038/s41593-021-00999-y. 35115731 PMC8825285

[ref162] Jang Y.; et al. Mass Spectrometry–Based Proteomics Analysis of Human Substantia Nigra From Parkinson’s Disease Patients Identifies Multiple Pathways Potentially Involved in the Disease. Mol. Cell. Proteom. 2023, 22, 100452 10.1016/j.mcpro.2022.100452. PMC9792365 36423813

[ref163] Davalieva K.; Kostovska I. M.; Dwork A. J. Proteomics Research in Schizophrenia. Front. Cell. Neurosci. 2016, 10, 18 10.3389/fncel.2016.00018. 26909022 PMC4754401

[ref164] Martins-de-Souza D.; et al. Proteome analysis of the thalamus and cerebrospinal fluid reveals glycolysis dysfunction and potential biomarkers candidates for schizophrenia. J. Psychiatr. Res. 2010, 44, 1176–1189. 10.1016/j.jpsychires.2010.04.014. 20471030

[ref165] Han M. H.; et al. Proteomic analysis of active multiple sclerosis lesions reveals therapeutic targets. Nature 2008, 451, 1076–1081. 10.1038/nature06559. 18278032

[ref166] Sandi D.; et al. Proteomics in Multiple Sclerosis: The Perspective of the Clinician. Int. J. Mol. Sci. 2022, 23, 5162 10.3390/ijms23095162. 35563559 PMC9100097

[ref167] Craft G. E.; Chen A.; Nairn A. C. Recent advances in quantitative neuroproteomics. Methods 2013, 61, 186–218. 10.1016/j.ymeth.2013.04.008. 23623823 PMC3891841

[ref168] Li K. W.; Ganz A. B.; Smit A. B. Proteomics of neurodegenerative diseases: analysis of human post-mortem brain. J. Neurochem. 2019, 151, 435–445. 10.1111/jnc.14603. 30289976 PMC6899881

[ref169] Yu Q.; et al. Isobaric Labeling Strategy Utilizing 4-Plex N,N-Dimethyl Leucine (DiLeu) Tags Reveals Proteomic Changes Induced by Chemotherapy in Cerebrospinal Fluid of Children with B-Cell Acute Lymphoblastic Leukemia. J. Proteome Res. 2020, 19, 2606–2616. 10.1021/acs.jproteome.0c00291. 32396724 PMC7334086

[ref170] Zhang X.; et al. Quantitative proteomic analysis of serum proteins in patients with Parkinson’s disease using an isobaric tag for relative and absolute quantification labeling, two-dimensional liquid chromatography, and tandem mass spectrometry. Analyst 2012, 137, 490–495. 10.1039/C1AN15551B. 22108571

[ref171] Dumrongprechachan V.; Salisbury R. B.; Butler L.; MacDonald M. L.; Kozorovitskiy Y. Dynamic proteomic and phosphoproteomic atlas of corticostriatal axons in neurodevelopment. eLife 2022, 11, e78847 10.7554/eLife.78847. 36239373 PMC9629834

[ref172] Xu B.; et al. Protein profile changes in the frontotemporal lobes in human severe traumatic brain injury. Brain Res. 2016, 1642, 344–352. 10.1016/j.brainres.2016.04.008. 27067185

[ref173] Rao-Ruiz P.; et al. Time-dependent changes in the mouse hippocampal synaptic membrane proteome after contextual fear conditioning. Hippocampus 2015, 25, 1250–1261. 10.1002/hipo.22432. 25708624

[ref174] Doll S.; Burlingame A. L. Mass Spectrometry-Based Detection and Assignment of Protein Posttranslational Modifications. ACS Chem. Biol. 2015, 10, 63–71. 10.1021/cb500904b. 25541750 PMC4301092

[ref175] Leutert M.; Entwisle S. W.; Villén J. Decoding Post-Translational Modification Crosstalk With Proteomics. Mol. Cell. Proteom. 2021, 20, 100129 10.1016/j.mcpro.2021.100129. PMC8430371 34339852

[ref176] Sanford E. J.; Smolka M. B. A field guide to the proteomics of post-translational modifications in DNA repair. PROTEOMICS 2022, 22, e2200064 10.1002/pmic.202200064. 35695711 PMC9950963

[ref177] Lee J. M.; Hammarén H. M.; Savitski M. M.; Baek S. H. Control of protein stability by post-translational modifications. Nat. Commun. 2023, 14, 201 10.1038/s41467-023-35795-8. 36639369 PMC9839724

[ref178] Thygesen C.; Boll I.; Finsen B.; Modzel M.; Larsen M. R. Characterizing disease-associated changes in post-translational modifications by mass spectrometry. Expert Rev. Proteom. 2018, 15, 245–258. 10.1080/14789450.2018.1433036. 29376447

[ref179] Mnatsakanyan R.; et al. Detecting post-translational modification signatures as potential biomarkers in clinical mass spectrometry. Expert Rev. Proteom. 2018, 15, 515–535. 10.1080/14789450.2018.1483340. 29893147

[ref180] Groban E. S.; Narayanan A.; Jacobson M. P. Conformational Changes in Protein Loops and Helices Induced by Post-Translational Phosphorylation. PLoS Comput. Biol. 2006, 2, e32 10.1371/journal.pcbi.0020032. 16628247 PMC1440919

[ref181] Ardito F.; Giuliani M.; Perrone D.; Troiano G.; Muzio L. L. The crucial role of protein phosphorylation in cell signaling and its use as targeted therapy (Review). Int. J. Mol. Med. 2017, 40, 271–280. 10.3892/ijmm.2017.3036. 28656226 PMC5500920

[ref182] Dephoure N.; Gould K. L.; Gygi S. P.; Kellogg D. R. Mapping and analysis of phosphorylation sites: a quick guide for cell biologists. Mol. Biol. Cell 2013, 24, 535–542. 10.1091/mbc.e12-09-0677. 23447708 PMC3583658

[ref183] Zhang G.; Neubert T. A. Phospho-Proteomics, Methods and Protocols. Methods Mol. Biol. 2009, 527, 79–92. 10.1007/978-1-60327-834-8_7. 19241007 PMC3757925

[ref184] Molden R. C.; Goya J.; Khan Z.; Garcia B. A. Stable Isotope Labeling of Phosphoproteins for Large-scale Phosphorylation Rate Determination*. Mol. Cell. Proteom. 2014, 13, 1106–1118. 10.1074/mcp.O113.036145. PMC3977188 24532841

[ref185] Hogrebe A.; et al. Benchmarking common quantification strategies for large-scale phosphoproteomics. Nat. Commun. 2018, 9, 1045 10.1038/s41467-018-03309-6. 29535314 PMC5849679

[ref186] Fíla J.; Honys D. Enrichment techniques employed in phosphoproteomics. Amino Acids 2012, 43, 1025–1047. 10.1007/s00726-011-1111-z. 22002794 PMC3418503

[ref187] Jiang X.; et al. Sensitive and Accurate Quantitation of Phosphopeptides Using TMT Isobaric Labeling Technique. J. Proteome Res. 2017, 16, 4244–4252. 10.1021/acs.jproteome.7b00610. 29022350

[ref188] Ohtsubo K.; Marth J. D. Glycosylation in Cellular Mechanisms of Health and Disease. Cell 2006, 126, 855–867. 10.1016/j.cell.2006.08.019. 16959566

[ref189] Varki A. Biological roles of glycans. Glycobiology 2017, 27, 3–49. 10.1093/glycob/cww086. 27558841 PMC5884436

[ref190] Yan A.; Lennarz W. J. Unraveling the Mechanism of Protein N-Glycosylation*. J. Biol. Chem. 2005, 280, 3121–3124. 10.1074/jbc.R400036200. 15590627

[ref191] Schachter H. The joys of HexNAc. The synthesis and function of N-andO-glycan branches. Glycoconj. J. 2000, 17, 465–483. 10.1023/A:1011010206774. 11421343

[ref192] Pan S.; Chen R.; Aebersold R.; Brentnall T. A. Mass Spectrometry Based Glycoproteomics—From a Proteomics Perspective*. Mol. Cell. Proteom. 2011, 10, R110.003251 10.1074/mcp.R110.003251. PMC3013464 20736408

[ref193] Alpert A. J. Hydrophilic-interaction chromatography for the separation of peptides, nucleic acids and other polar compounds. J. Chromatogr. A 1990, 499, 177–196. 10.1016/S0021-9673(00)96972-3. 2324207

[ref194] Hägglund P.; Bunkenborg J.; Elortza F.; Jensen O. N.; Roepstorff P. A New Strategy for Identification of N-Glycosylated Proteins and Unambiguous Assignment of Their Glycosylation Sites Using HILIC Enrichment and Partial Deglycosylation. J. Proteome Res. 2004, 3, 556–566. 10.1021/pr034112b. 15253437

[ref195] Wang D.; et al. Boost-DiLeu: Enhanced Isobaric N,N-Dimethyl Leucine Tagging Strategy for a Comprehensive Quantitative Glycoproteomic Analysis. Anal. Chem. 2022, 94, 11773–11782. 10.1021/acs.analchem.2c01773. 35960654 PMC9966376

[ref196] Mondal S.; Thompson P. R. Protein Arginine Deiminases (PADs): Biochemistry and Chemical Biology of Protein Citrullination. Acc. Chem. Res. 2019, 52, 818–832. 10.1021/acs.accounts.9b00024. 30844238 PMC6443095

[ref197] Darrah E.; Andrade F. Rheumatoid arthritis and citrullination. Curr. Opin. Rheumatol. 2018, 30, 72–78. 10.1097/BOR.0000000000000452. 28937414 PMC5848217

[ref198] Kuhn K. A.; et al. Antibodies against citrullinated proteins enhance tissue injury in experimental autoimmune arthritis. J. Clin. Investig. 2006, 116, 961–973. 10.1172/JCI25422. 16585962 PMC1421345

[ref199] Moscarello M. A.; Mastronardi F. G.; Wood D. D. The Role of Citrullinated Proteins Suggests a Novel Mechanism in the Pathogenesis of Multiple Sclerosis. Neurochem. Res. 2007, 32, 251–256. 10.1007/s11064-006-9144-5. 17031564 PMC1794624

[ref200] Ishigami A.; et al. Abnormal accumulation of citrullinated proteins catalyzed by peptidylarginine deiminase in hippocampal extracts from patients with Alzheimer’s disease. J. Neurosci. Res. 2005, 80, 120–128. 10.1002/jnr.20431. 15704193

[ref201] Yuzhalin A. E. Citrullination in Cancer. Cancer Res. 2019, 79, 1274–1284. 10.1158/0008-5472.CAN-18-2797. 30894374

[ref202] Clancy K. W.; Weerapana E.; Thompson P. R. Detection and identification of protein citrullination in complex biological systems. Curr. Opin. Chem. Biol. 2016, 30, 1–6. 10.1016/j.cbpa.2015.10.014. 26517730 PMC4731267

[ref203] Rebak A. S.; Hendriks I. A.; Nielsen M. L. Characterizing citrullination by mass spectrometry-based proteomics. Philos. Trans. R. Soc. B 2023, 378, 20220237 10.1098/rstb.2022.0237. PMC10542455 37778389

[ref204] Holm A.; et al. Specific modification of peptide-bound citrulline residues. Anal. Biochem. 2006, 352, 68–76. 10.1016/j.ab.2006.02.007. 16540076

[ref205] Li Z.; Wang B.; Yu Q.; Shi Y.; Li L. 12-Plex DiLeu Isobaric Labeling Enabled High-Throughput Investigation of Citrullination Alterations in the DNA Damage Response. Anal. Chem. 2022, 94, 3074–3081. 10.1021/acs.analchem.1c04073. 35129972 PMC9055876

[ref206] Yu C.; Huang L. Cross-Linking Mass Spectrometry: An Emerging Technology for Interactomics and Structural Biology. Anal. Chem. 2018, 90, 144–165. 10.1021/acs.analchem.7b04431. 29160693 PMC6022837

[ref207] Orbán-Németh Z.; et al. Structural prediction of protein models using distance restraints derived from cross-linking mass spectrometry data. Nat. Protoc. 2018, 13, 478–494. 10.1038/nprot.2017.146. 29419816 PMC5999019

[ref208] Politis A.; Schmidt C. Structural characterisation of medically relevant protein assemblies by integrating mass spectrometry with computational modelling. J. Proteom. 2018, 175, 34–41. 10.1016/j.jprot.2017.04.019. 28461040

[ref209] Degiacomi M. T.; Schmidt C.; Baldwin A. J.; Benesch J. L. P. Accommodating Protein Dynamics in the Modeling of Chemical Crosslinks. Structure 2017, 25, 1751–1757. 10.1016/j.str.2017.08.015. 28966018

[ref210] Yu C.; et al. Developing a Multiplexed Quantitative Cross-Linking Mass Spectrometry Platform for Comparative Structural Analysis of Protein Complexes. Anal. Chem. 2016, 88, 10301–10308. 10.1021/acs.analchem.6b03148. 27626298 PMC5361889

[ref211] Yu C.; Wang X.; Huang L. Developing a Targeted Quantitative Strategy for Sulfoxide-Containing MS-Cleavable Cross-Linked Peptides to Probe Conformational Dynamics of Protein Complexes. Anal. Chem. 2022, 94, 4390–4398. 10.1021/acs.analchem.1c05298. 35193351 PMC9000990

[ref212] Wippel H. H.; Chavez J. D.; Tang X.; Bruce J. E. Quantitative interactome analysis with chemical cross-linking and mass spectrometry. Curr. Opin. Chem. Biol. 2022, 66, 102076 10.1016/j.cbpa.2021.06.011. 34393043 PMC8837725

[ref213] Chen Z. A.; Rappsilber J. Protein Dynamics in Solution by Quantitative Crosslinking/Mass Spectrometry. Trends Biochem. Sci. 2018, 43, 908–920. 10.1016/j.tibs.2018.09.003. 30318267 PMC6240160

[ref214] Ruwolt M.; et al. Optimized TMT-Based Quantitative Cross-Linking Mass Spectrometry Strategy for Large-Scale Interactomic Studies. Anal. Chem. 2022, 94, 5265–5272. 10.1021/acs.analchem.1c04812. 35290030

[ref215] Chavez J. D.; Keller A.; Mohr J. P.; Bruce J. E. Isobaric Quantitative Protein Interaction Reporter Technology for Comparative Interactome Studies. Anal. Chem. 2020, 92, 14094–14102. 10.1021/acs.analchem.0c03128. 32969639 PMC7995634

[ref216] Wippel H. H.; Chavez J. D.; Keller A. D.; Bruce J. E. Multiplexed Isobaric Quantitative Cross-Linking Reveals Drug-Induced Interactome Changes in Breast Cancer Cells. Anal. Chem. 2022, 94, 2713–2722. 10.1021/acs.analchem.1c02208. 35107270 PMC8969885

[ref217] Kelly R. T. Single-cell Proteomics: Progress and Prospects. Mol. Cell. Proteom. 2020, 19, 1739–1748. 10.1074/mcp.R120.002234. PMC7664119 32847821

[ref218] Gavasso S.; Gullaksen S.-E.; Skavland J.; Gjertsen B. T. Single-cell proteomics: potential implications for cancer diagnostics. Expert Rev. Mol. Diagn. 2016, 16, 579–589. 10.1586/14737159.2016.1156531. 26895397

[ref219] Wang H.; et al. Integrated Proteomics and Single-Cell Mass Cytometry Analysis Dissects the Immune Landscape of Ankylosing Spondylitis. Anal. Chem. 2023, 95, 7702–7714. 10.1021/acs.analchem.3c00809. 37126452

[ref220] Portero E. P.; Pade L. R.; Li J.; Choi S. B.; Nemes P. Single Cell ‘Omics of Neuronal Cells. Neuromethods 2022, 184, 87–114. 10.1007/978-1-0716-2525-5_5. 36699808 PMC9872963

[ref221] Boekweg H.; Payne S. H. Challenges and Opportunities for Single-cell Computational Proteomics. Mol. Cell. Proteom. 2023, 22, 100518 10.1016/j.mcpro.2023.100518. PMC10060113 36828128

[ref222] Lu H.; Zhang H.; Li L. Chemical tagging mass spectrometry: an approach for single-cell omics. Anal. Bioanal. Chem. 2023, 415, 6901–6913. 10.1007/s00216-023-04850-0. 37466681 PMC10729908

[ref223] Matzinger M.; Müller E.; Dürnberger G.; Pichler P.; Mechtler K. Robust and Easy-to-Use One-Pot Workflow for Label-Free Single-Cell Proteomics. Anal. Chem. 2023, 95, 4435–4445. 10.1021/acs.analchem.2c05022. 36802514 PMC9996606

[ref224] Bennett H. M.; Stephenson W.; Rose C. M.; Darmanis S. Single-cell proteomics enabled by next-generation sequencing or mass spectrometry. Nat. Methods 2023, 20, 363–374. 10.1038/s41592-023-01791-5. 36864196

[ref225] Schoof E. M.; et al. Quantitative single-cell proteomics as a tool to characterize cellular hierarchies. Nat. Commun. 2021, 12, 3341 10.1038/s41467-021-23667-y. 34099695 PMC8185083

[ref226] Lee S.; Vu H. M.; Lee J.-H.; Lim H.; Kim M.-S. Advances in Mass Spectrometry-Based Single Cell Analysis. Biology 2023, 12, 395 10.3390/biology12030395. 36979087 PMC10045136

[ref227] Ye Z.; Batth T. S.; Rüther P.; Olsen J. V. A deeper look at carrier proteome effects for single-cell proteomics. Commun. Biol. 2022, 5, 150 10.1038/s42003-022-03095-4. 35194133 PMC8863851

[ref228] Messner C. B.; et al. Ultra-fast proteomics with Scanning SWATH. Nat. Biotechnol. 2021, 39, 846–854. 10.1038/s41587-021-00860-4. 33767396 PMC7611254

[ref229] Bekker-Jensen D. B.; et al. An Optimized Shotgun Strategy for the Rapid Generation of Comprehensive Human Proteomes. Cell Syst. 2017, 4, 587–599. 10.1016/j.cels.2017.05.009. 28601559 PMC5493283

[ref230] Derks J.; et al. Increasing the throughput of sensitive proteomics by plexDIA. Nat. Biotechnol. 2023, 41, 50–59. 10.1038/s41587-022-01389-w. 35835881 PMC9839897

[ref231] Schweppe D. K.; et al. Full-Featured, Real-Time Database Searching Platform Enables Fast and Accurate Multiplexed Quantitative Proteomics. J. Proteome Res. 2020, 19, 2026–2034. 10.1021/acs.jproteome.9b00860. 32126768 PMC7295121

[ref232] Furtwängler B.; et al. Real-Time Search-Assisted Acquisition on a Tribrid Mass Spectrometer Improves Coverage in Multiplexed Single-Cell Proteomics. Mol. Cell. Proteom. 2022, 21, 100219 10.1016/j.mcpro.2022.100219. PMC8961214 35219906

[ref233] Yu Q.; et al. Sample multiplexing-based targeted pathway proteomics with real-time analytics reveals the impact of genetic variation on protein expression. Nat. Commun. 2023, 14, 555 10.1038/s41467-023-36269-7. 36732331 PMC9894840

[ref234] Scientific Image and Illustration Software. BioRender. Figure created with BioRender.com. https://www.biorender.com/ (March 29, 2024).

[ref235] Overmyer K. A.; et al. Multiplexed proteome analysis with neutron-encoded stable isotope labeling in cells and mice. Nat. Protoc. 2018, 13, 293–306. 10.1038/nprot.2017.121. 29323663 PMC5920564

